# Cucumber Biomass Powder as Reinforcing Scaffold for Poly(acrylic acid-co-acrylamide) Superabsorbent Hydrogels: Structure and Properties

**DOI:** 10.3390/gels12090768

**Published:** 2026-08-27

**Authors:** Yuliang Lu, Jianping He, Yichun Zeng, Tianyu Liao, Lezi Li, Chenfei Xu, Yi Shi, Jiaoning Tang, Yang He

**Affiliations:** 1 School of Materials and New Energy, South China Normal University, Shanwei 516600, China; 20238031028@m.scnu.edu.cn (Y.L.); 2024025510@m.scnu.edu.cn (J.H.); 20238021046@m.scnu.edu.cn (Y.Z.); 20238031058@m.scnu.edu.cn (T.L.); 202480313895@m.scnu.edu.cn (L.L.); 2023025268@m.scnu.edu.cn (C.X.); tjn@szu.edu.cn (J.T.); 2 School of Materials Science and Engineering, Sun Yat-Sen University, Guangzhou 510006, China

**Keywords:** superabsorbent polymer, cucumber, hydrogel, biomass, rheology, hydrogen bonding, saline responsiveness

## Abstract

Unrefined cucumber biomass powder (CBP) was incorporated into a poly(acrylic acid-co-acrylamide) (P(AA-co-AM)) network to develop a superabsorbent hydrogel with enhanced gel network rigidity and swelling performance. CBP served as a renewable reinforcing scaffold, providing a multicomponent biomass matrix for physical reinforcement alongside abundant hydrophilic groups (–OH, –NH, and –CONH–). Spectroscopic and rheological analyses suggest that CBP integration significantly alters the gel network through complex multi-component interactions, as evidenced by FTIR peak shifts (3435 → 3442 cm^−1^, 1558 → 1567 cm^−1^) that are consistent with extensive interfacial hydrogen bonding and potential partial chemical integration, leading to a 97% increase in storage modulus (from 11.9 kPa to 23.4 kPa) with a corresponding decrease in tanδ from 0.36 to 0.26. Under optimized synthesis conditions, the composite exhibited 30 min free-swelling capacities of 2801.00 g/g in deionized water, 142.72 g/g in 0.9 wt% NaCl, and 65.04 g/g in synthetic urine, along with rapid kinetics (1637.49 g/g within 1 min) and partial residual swelling capacity (727.44 g/g retained after five cycles). Rheological analysis revealed that CBP incorporation enhances network stiffness while introducing a more strain-sensitive response at high deformations—a typical trade-off for rigidized gel networks. These findings demonstrate that unrefined model biomass can serve as an effective, low-cost reinforcing scaffold for functional hydrogels, offering a sustainable route for personal care or agricultural applications in gel materials design.

## 1. Introduction

Superabsorbent polymers (SAPs) can rapidly absorb and retain large amounts of liquid, making them essential materials for commercial absorbent cores, incontinence products, and other personal care and related applications [1,2,3,4]. Notably, advanced hydrogel networks have also shown tremendous application potential in the fields of tissue engineering and bioprinted repair materials [5]. Furthermore, driven by the dual requirements of high performance and sustainability, superabsorbent hydrogels based on natural biomass (especially cellulose and its derivatives) have become a major research hotspot in the current materials science community [6,7]. Compared with agricultural water retention, personal care and related applications often involve saline-containing body fluids (e.g., urine and physiological saline). Such systems require not only high absorption capacity but also appreciable absorption performance in saline environments, fast liquid uptake, robust gel integrity to prevent structural failure under stress, resistance to liquid loss under external pressure (absorption under load, AUL) and gravitational forces (liquid retention), as well as thorough safety evaluations to ensure biocompatibility and the absence of harmful residuals.

Current commercial SAPs are mainly derived from petroleum-based polyacrylate polymers. They exhibit excellent water absorption properties and mature manufacturing processes, but concerns have been raised regarding sustainability and end-of-life disposal. On the one hand, petroleum-based feedstocks are non-renewable and difficult to degrade; on the other hand, the large consumption of such materials in commercial absorbent products makes the environmental burden across the whole life cycle a non-negligible issue [8]. Driven by the dual requirements of high performance and sustainability, natural-biomass-based superabsorbent hydrogels have become a research hotspot [9,10]. Natural polysaccharides and their composite systems (e.g., cellulose, pectin, hemicellulose) possess abundant hydroxyl groups (particularly in cellulose and pectin) and polysaccharide backbones, showing good hydrophilicity and potential biocompatibility. Meanwhile, their fibrous or particulate morphology can serve as multi-scale pore channels and network scaffolds, which may improve the structural stability and absorption kinetics of the resulting gels [11,12]. Nevertheless, biomass-based materials generally face two key bottlenecks. First, their absorption in saline solutions deteriorates significantly because of ion screening and decreased osmotic pressure [13,14]. Second, the large swelling tends to be accompanied by reduced gel strength, structural breakdown or leakage after liquid uptake, which limits their reliable application in real care scenarios [15]. Therefore, a core scientific issue for translating such materials into practical applications is how to improve saline absorption and absorption rate while maintaining gel water retention through rational network design and synergistic use of chemical groups.

Model biomass provides a renewable source for the development of green SAPs. Cucumber is widely cultivated, has a short growth cycle and high yield, and its tissues are rich in polysaccharide components (such as cellulose-related backbones and other polysaccharides), providing a natural basis for constructing hydrophilic networks [16]. The cucumber biomass powder (CBP) used in this work is an unrefined, whole-component plant powder. The chemical composition of CBP was analyzed by proximate analysis. The results were as follows: crude protein 17.7%, crude fat 3.5%, crude fiber 6.2%, moisture 11.0%, and crude ash 8.9%. The remaining fraction (approximately 52.7%) was assigned to nitrogen-free extract (NFE), which mainly consists of soluble sugars, pectin, hemicellulose, and other carbohydrates. Since these components are rich in -OH groups and also contain some -COOH groups, they may interact with the P(AA-co-AM) network through hydrogen bonding or ionic interactions, potentially enhancing both the absorption kinetics and the liquid-retention stability of the material, thereby providing a basis for constructing low-cost, circular absorption systems.

Based on the above considerations, a P(AA-co-AM)/CBP composite superabsorbent hydrogel was prepared via free-radical polymerization in aqueous solution. For comparison, the P(AA-co-AM) SAP without CBP was also prepared under the same conditions. The chemical structure, aggregation state, morphology, and thermal stability of the hydrogels were characterized by FTIR, XRD, SEM, and TGA. Their absorption capacity, swelling kinetics, water retention, cyclic swelling behavior, centrifuge retention capacity, absorption under load (AUL), and rheological properties were systematically evaluated. Through a combination of structural and macroscopic analyses, the primary objective of this study is to systematically investigate the effect of unrefined CBP incorporation on the properties of the P(AA-co-AM) network. Rather than presuming pre-established covalent grafting or rigid reinforcement mechanisms, this work aims to explore how the dispersion of the multicomponent biomass matrix within the polymer network, together with the interfacial interactions (such as extensive hydrogen bonding) between them, governs the overall gel network structure, swelling behavior, and mechanical performance. Ultimately, the goal is to evaluate the feasibility of utilizing unrefined model biomass in the development of bio-based superabsorbent materials. This strategy highlights the immense potential of utilizing low-cost, widely available cucumber biomass to achieve high-value-added applications in the development of green, circular, bio-based superabsorbent materials.

## 2. Results and Discussion

### 2.1. Synthesis and Swelling Mechanism

**Figure 1:**
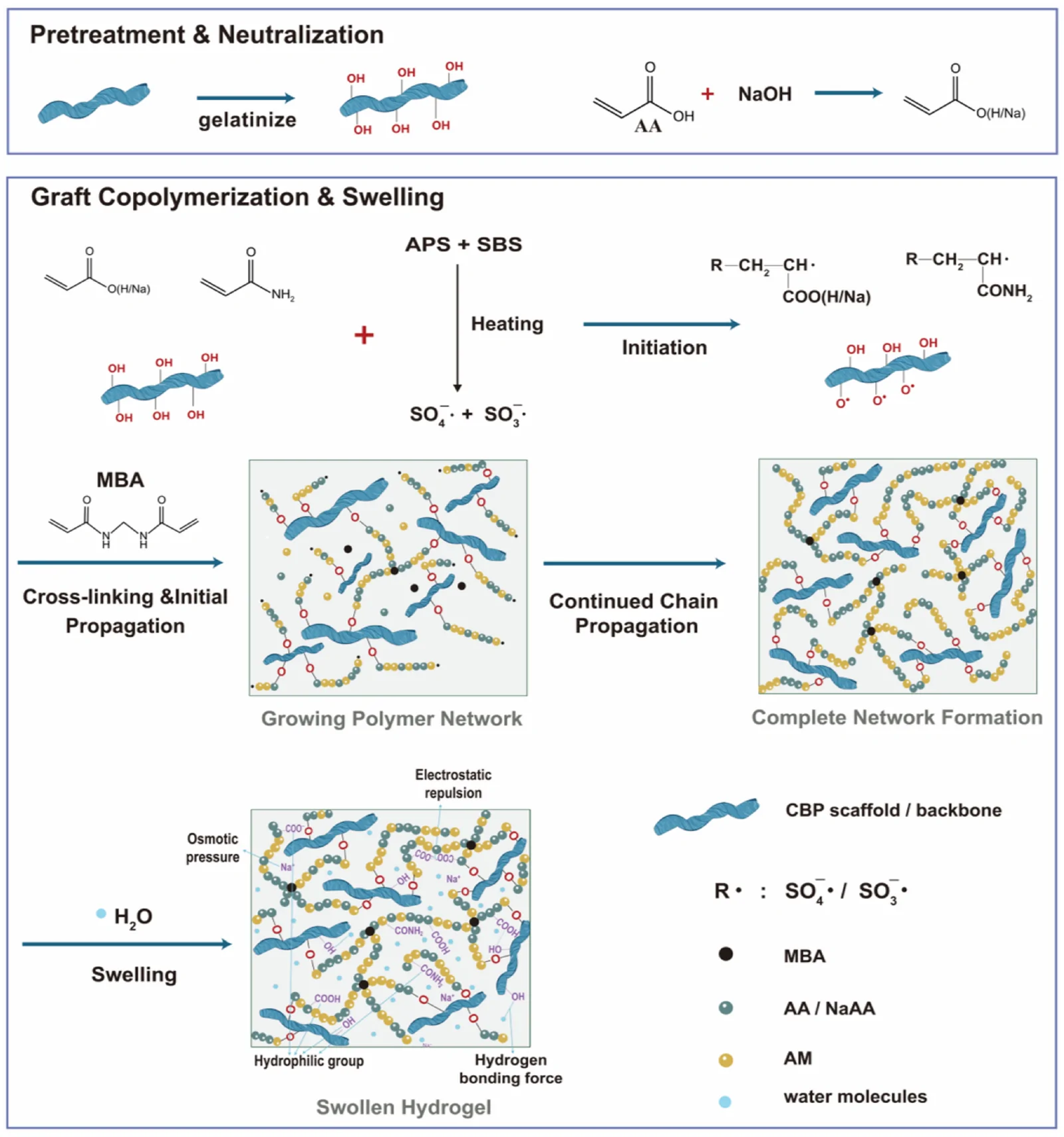
Schematic illustration of the synthesis and swelling mechanism of P(AA-co-AM)/CBP SAP.

The P(AA-co-AM)/CBP composite hydrogel was designed to combine the rigid scaffold of CBP with a chemically crosslinked polyelectrolyte network. Figure 1 schematically illustrates the proposed synthesis route and the underlying swelling mechanism.

#### 2.1.1. Synthesis Mechanism

As illustrated in Figure 1, the synthesis of P(AA-co-AM)/CBP SAP involves the following key steps. In the gelatinization and pretreatment stage, the cucumber biomass powder (CBP) dispersed in the aqueous phase undergoes thorough hydration and swelling under constant-temperature heating and stirring, thereby exposing abundant active hydroxyl (-OH) sites on the surface of its polysaccharide backbone. Meanwhile, acrylic acid (AA) is partially neutralized with sodium hydroxide to generate a mixture containing sodium acrylate, providing the basis for the subsequent construction of a highly absorbent network. Subsequently, the neutralized AA, acrylamide (AM) monomers, and the crosslinker (MBA) are uniformly dispersed in the aqueous phase surrounding the CBP backbone.

During the initiation stage (Figure 1), the redox initiator system composed of ammonium persulfate (APS) and sodium bisulfite (SBS) decomposes under heating to produce highly active primary free radicals such as sulfate radical anions (SO_4_•^−^) [17]. A competitive dual initiation then occurs in the system. On the one hand, the primary radicals abstract hydrogen atoms from the exposed -OH groups on the CBP backbone, generating macro-oxygen radicals (-O•) on the polysaccharide backbone [18]. On the other hand, the primary radicals simultaneously attack the carbon–carbon double bonds of free AA (or NaAA) and AM monomers in the aqueous phase, forming highly active monomeric radicals.

In the chain growth and crosslinking stage, the macro-oxygen radicals (-O•) on the CBP backbone act as highly active initiating sites. They continuously capture and initiate the nearby free AA (or NaAA) and AM monomers via double-bond addition, leading to the in situ growth of P(AA-co-AM) random copolymer chains on the backbone. During this graft copolymerization and the simultaneous copolymerization of free monomers, the bifunctional crosslinker N,N′-methylenebisacrylamide (MBA) participates in the reaction. Through its two-terminal carbon–carbon double bonds, MBA chemically crosslinks the adjacent growing P(AA-co-AM) chains, including both those grafted onto CBP and those free in solution. Ultimately, this polymerization process successfully constructs a hydrogel network with a stable three-dimensional structure, in which the CBP component serves as a tough structural support and water-conducting backbone [19]. It must be emphasized that the mechanical reinforcement of the hydrogel network by unrefined CBP is not solely due to a single cellulose backbone, but originates from its inherent multicomponent complexity. The extensive hemicellulose, pectin, small amounts of crude protein, and soluble sugars present in CBP contain abundant active hydrophilic functional groups, such as hydroxyl, carboxyl, and amino groups. These natural components work synergistically in the aqueous phase to form a dense hydrogen bonding network and physical entanglements with the polymer chains. Therefore, CBP actually functions as a multicomponent synergistic natural biomass matrix, integrally participating in the reinforcement of the supramolecular network.

It should be explicitly noted that the reaction pathway described above represents the proposed theoretical mechanism. While the generation of macro-oxygen radicals on the CBP surface and subsequent in situ chain growth are well documented in classical free-radical polymerization of biomass systems, the absolute quantification of covalent grafting efficiency requires rigorous extraction and gel-fraction analyses. In the absence of such quantitative techniques in this study, the structural reinforcement of the composite network is strictly attributed to a synergistic combination of extensive interfacial hydrogen bonding, physical entanglement with the multicomponent biomass matrix, and potential partial chemical integration, rather than solely relying on confirmed covalent grafting.

#### 2.1.2. Swelling Mechanism

When the dried SAP powder comes into contact with water, a hydration layer rapidly forms on its surface due to the abundant hydrophilic groups present in the system, including the -OH groups from CBP, the -COOH/-COO^−^ groups from AA, and the -CONH_2_ groups from AM. Driven by capillary forces and concentration gradients, water molecules penetrate into the interior of the particles through surface pores and interparticle voids. Meanwhile, the original polymer–polymer hydrogen bonds within the network are gradually replaced by polymer–water hydrogen bonds, which enhance chain flexibility and increases free volume, thereby further accelerating water absorption and swelling [20].

The primary driving force for the ultra-high swelling capacity of this composite network lies in the electrostatic repulsion among carboxylate anions and the Donnan osmotic pressure difference arising from the counterions. Because AA is partially neutralized before polymerization, a considerable proportion of -COONa groups exist in the network. During water absorption, these ionic groups dissociate, increasing the density of fixed negative charges (-COO^−^) along the polymer backbone. This generates a strong electrostatic repulsion within the network, promoting pore expansion [14]. At the same time, the concentration of mobile counterions (e.g., Na^+^) inside the gel is higher than that in the external solution, creating an osmotic pressure difference. Under this high osmotic pressure, a large number of external water molecules spontaneously and continuously enter the hydrogel network until the system reaches swelling equilibrium [21]. In addition, the -CONH_2_ groups introduced by the acrylamide (AM) segments further enhance the network hydrophilicity through dipole–water interactions and hydrogen bonding, thereby stabilizing the swollen state.

Owing to the finite crosslinking degree introduced by MBA and the constraining effect of the multicomponent biomass matrix, the crosslinked network of SAP does not swell indefinitely [19,20]. The system reaches its final swelling equilibrium when the expansion driving force from water influx is balanced by the elastic retraction force derived from the crosslinked network and the rigid scaffold. In this process, the untreated CBP acts as a natural polysaccharide scaffold. Its abundant -OH groups provide continuous water migration channels, while its multicomponent matrix not only suppresses excessive chain relaxation under high swelling, thereby maintaining the structural integrity of the swollen gel, but also endows the material with good cyclic swelling durability. The salt sensitivity of this composite will be discussed in Section 2.9 in comparison with commercial SAPs.

In summary, this study establishes a clear ‘structure–property’ correlation logic: the incorporation of CBP provides abundant interfacial hydrogen bonding sites and a rigid natural composite scaffold. This synergistic effect transforms the dense polymer matrix into a highly open, interconnected porous network. This specific structural evolution directly translates into the significant enhancement of macroscopic performance, including ultra-high water absorption capacity, rapid kinetic response, and excellent mechanical stiffness under load.

### 2.2. FTIR Analysis

**Figure 2:**
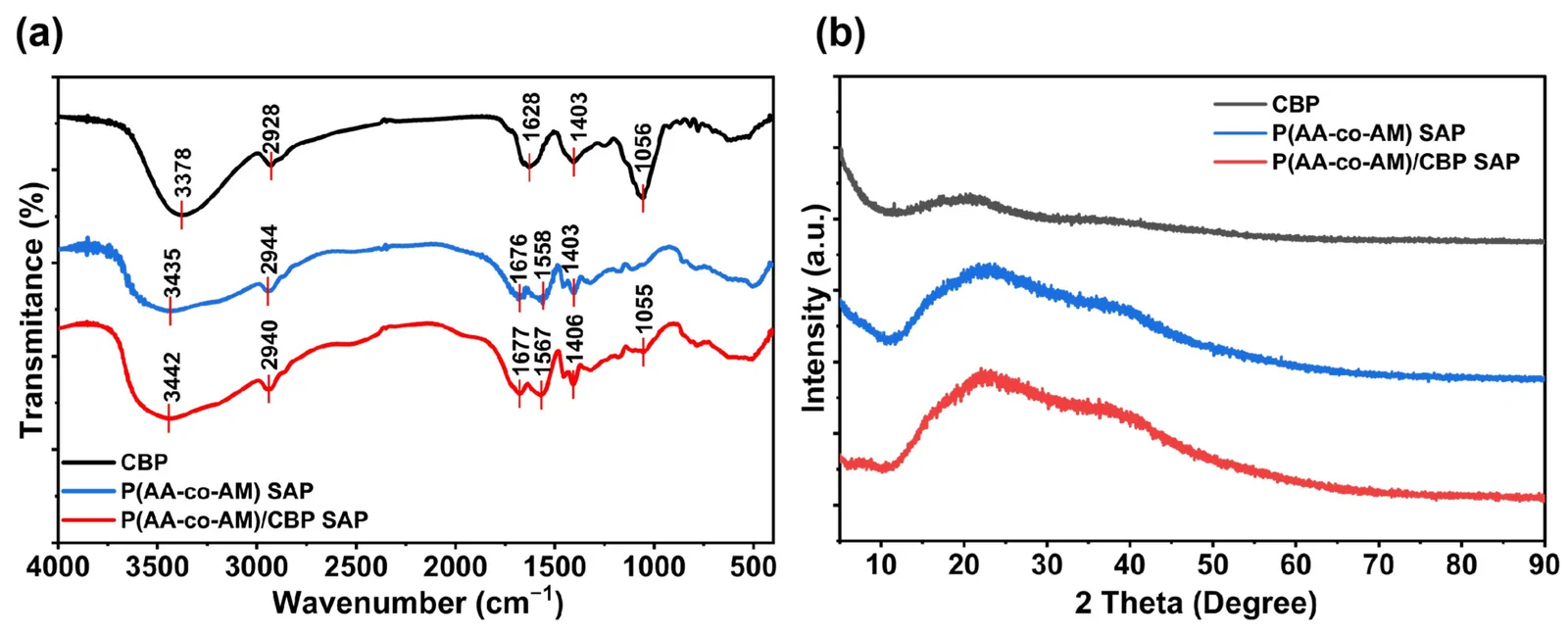
(**a**) FTIR spectra of CBP (**top**), P(AA-co-AM) SAP (**middle**), and P(AA-co-AM)/CBP SAP (**bottom**); (**b**) XRD patterns of CBP (**top**), P(AA-co-AM) SAP (**middle**), and P(AA-co-AM)/CBP SAP (**bottom**). The FTIR spectra in (**a**) and XRD patterns in (**b**) are each vertically shifted relative to each other for clarity.

The FTIR spectra of CBP, P(AA-co-AM) SAP, and P(AA-co-AM)/CBP SAP are shown in Figure 2a.

For CBP, the broad and strong absorption band at 3378 cm^−1^ is attributed to the -OH stretching vibration of polysaccharides and related hydrophilic components; the significant broadening results from abundant hydrogen bonding within the biomass. This band may also contain a contribution from the N-H stretching vibration (Amide A) of residual proteins. The absorption peak at 2928 cm^−1^ corresponds to the asymmetric C-H stretching vibration of -CH_2_- groups. The strong peak at 1056 cm^−1^ is assigned to the C-O-C or C-O stretching vibration of polysaccharide structures, a characteristic feature of plant-derived polysaccharide materials, indicating a clear polysaccharide backbone signature for this powder [22,23]. It should be noted that, because CBP is an unrefined, whole-component cucumber biomass powder, its FTIR spectrum also exhibits absorptions at 1628 cm^−1^ and 1403 cm^−1^. These peaks can be assigned to the amide I (C=O stretching) and amide II (N-H bending coupled with C-N stretching) vibrations of protein components, respectively, reflecting the complex multi-component nature of CBP.

In the spectrum of pure P(AA-co-AM) SAP, the broad band at 3435 cm^−1^ corresponds to the overlapping stretching vibrations of -OH and -NH groups. The peak at 2944 cm^−1^ is assigned to the saturated C-H stretching vibration of the polymer backbone. The strong and broad absorption at 1676 cm^−1^ is primarily attributed to the C=O stretching vibration of hydrogen-bonded carboxyl groups (-COOH), which may also contain a contribution from the amide I band (C=O stretching) of the AM units. The absorption peaks at 1558 cm^−1^ and 1403 cm^−1^ correspond to the asymmetric and symmetric stretching vibrations of -COO^−^ groups, respectively. Notably, while the peak at 1403 cm^−1^ in pure CBP is associated with the amide II band of proteins, in the pure SAP spectrum it is clearly assigned to the -COO^−^ symmetric stretching vibration, as no protein components are present in the synthetic polymer. In addition, the 1558 cm^−1^ region may also overlap with the amide II band, which is dominated by N-H in-plane bending coupled with C-N stretching. These features, especially the characteristic absorptions of carboxylate groups (asymmetric and symmetric stretching of -COO^−^), indicate the presence of ionized carboxylic acid species within the network, confirming the formation of a charged hydrophilic structure [24].

After the incorporation of CBP, the composite hydrogel retains the main characteristic absorption peaks of the P(AA-co-AM) network. Notably, a distinct absorption shoulder corresponding to the polysaccharide C-O-C or C-O stretching vibration is observed near 1055 cm^−1^, confirming the successful introduction of CBP into the composite system. Compared with pure P(AA-co-AM), the composite hydrogel exhibits subtle but consistent shifts in several characteristic bands: the -OH/-NH stretching band moves from 3435 cm^−1^ to 3442 cm^−1^, while the asymmetric -COO^−^ stretching band (partially overlapping with the amide II region) shifts from 1558 cm^−1^ to 1567 cm^−1^. These frequency shifts suggest the presence of specific intermolecular interactions, most likely hydrogen bonding, between the hydroxyl groups on the CBP surface and the -COOH, -COO^−^, or -CONH_2_ groups of the P(AA-co-AM) network. The formation of hydrogen bonds can alter the local chemical environment and modify the force constants of the corresponding bonds, thereby shifting their characteristic vibrational frequencies [23]. Similar hydrogen bonding interactions between biomass components and polymer networks have been reported in other composite systems [25].

To further confirm that the observed structural changes result from chemical grafting rather than simple physical mixing, a control experiment was performed using a physical mixture of CBP and P(AA-co-AM) SAP (1:10 wt/wt). The FTIR spectrum of the physical mixture (Figure S2, Supporting Information) shows the CBP-derived C-O-C band at 1046 cm^−1^, while the characteristic 776 cm^−1^ fingerprint band of P(AA-co-AM) remains unchanged. In contrast, the chemically synthesized composite exhibits the polysaccharide C-O-C band at approximately 1055 cm^−1^, which is markedly different from that of the physical mixture (1046 cm^−1^). This distinct difference in the C-O-C band position—coupled with the characteristic shifts in the -OH/-NH and -COO^−^ stretching regions described above [23]—provides spectroscopic evidence of significant molecular-level interactions that differentiate the synthesized composite from a simple physical mixture. However, it must be acknowledged that the physical-mixture control was prepared via simple dry blending and did not undergo the same aqueous neutralization, thermal polymerization, cyclic anhydrous ethanol washing, and rigorous drying procedures as the synthesized composite. While absolute quantification of covalent grafting would require complex solid-state techniques, these distinct vibrational peak shifts cannot be attributed exclusively to covalent grafting. Rather, they strongly reflect a combination of the extensive reorganization of interfacial hydrogen bonds induced by the wet synthesis history and the potential partial chemical integration of the CBP scaffold within the polymer network.

Consequently, the FTIR results demonstrate that CBP is not merely physically mixed but deeply incorporated into the P(AA-co-AM) network, with clear evidence of altered intermolecular interactions (see Figure S2).

### 2.3. XRD Analysis

The XRD patterns of CBP, P(AA-co-AM) SAP, and P(AA-co-AM)/CBP SAP are shown in Figure 2b.

CBP exhibits a broad diffraction peak at 2θ ≈ 21.0°, indicating a certain degree of short-range order, with the overall pattern dominated by an amorphous phase accompanied by weakly ordered regions. This diffraction feature is consistent with the typical 2θ range (~21–23°) reported for cellulose-containing biomass materials [26]. P(AA-co-AM) SAP displays a broad diffuse peak near 2θ ≈ 23.0° and a weak diffuse signal in the range of approximately 35–40°, indicating that the polymer network is predominantly amorphous. This is consistent with previous XRD studies on poly(acrylic acid-co-acrylamide) hydrogels, which have demonstrated their amorphous nature [27].

Compared with pure P(AA-co-AM), the main diffuse peak of P(AA-co-AM)/CBP SAP remains broad but shifts slightly to 2θ ≈ 22.3°, with further broadening of the peak shape. This suggests that the introduction of CBP does not create a new crystalline phase; instead, it perturbs the original local ordered packing of the polymer chains, driving the system toward a more disordered structure. It has been reported that in polysaccharide-containing acrylic acid/acrylamide composite network systems, the polymerization and crosslinking processes usually weaken the original crystalline regions and promote a transition to an amorphous structure [28]. These results indicate that during the free-radical polymerization, strong intermolecular interactions occur between the polysaccharide and other biomass components in CBP and the P(AA-co-AM) network, transforming the system from locally ordered packing to a looser, more disordered three-dimensional crosslinked structure. The absence of sharp diffraction peaks in the composite sample indicates that no distinct independent crystalline phases or large-scale crystalline aggregates are formed. In general, such an amorphous-network-dominated structure is favorable for increasing free volume, exposing more hydrophilic sites, and promoting water diffusion [29], which is consistent with the high absorption capacity and fast absorption rate observed for this material.

Combining the FTIR and XRD results, it can be concluded that CBP has been successfully introduced into the P(AA-co-AM) hydrogel network and that significant interactions exist between CBP and the polymer chains. These interactions are consistent with extensive hydrogen bonding and potential partial chemical integration, which facilitate the effective incorporation of the biomass into the hydrogel network.

### 2.4. SEM Analysis

**Figure 3:**
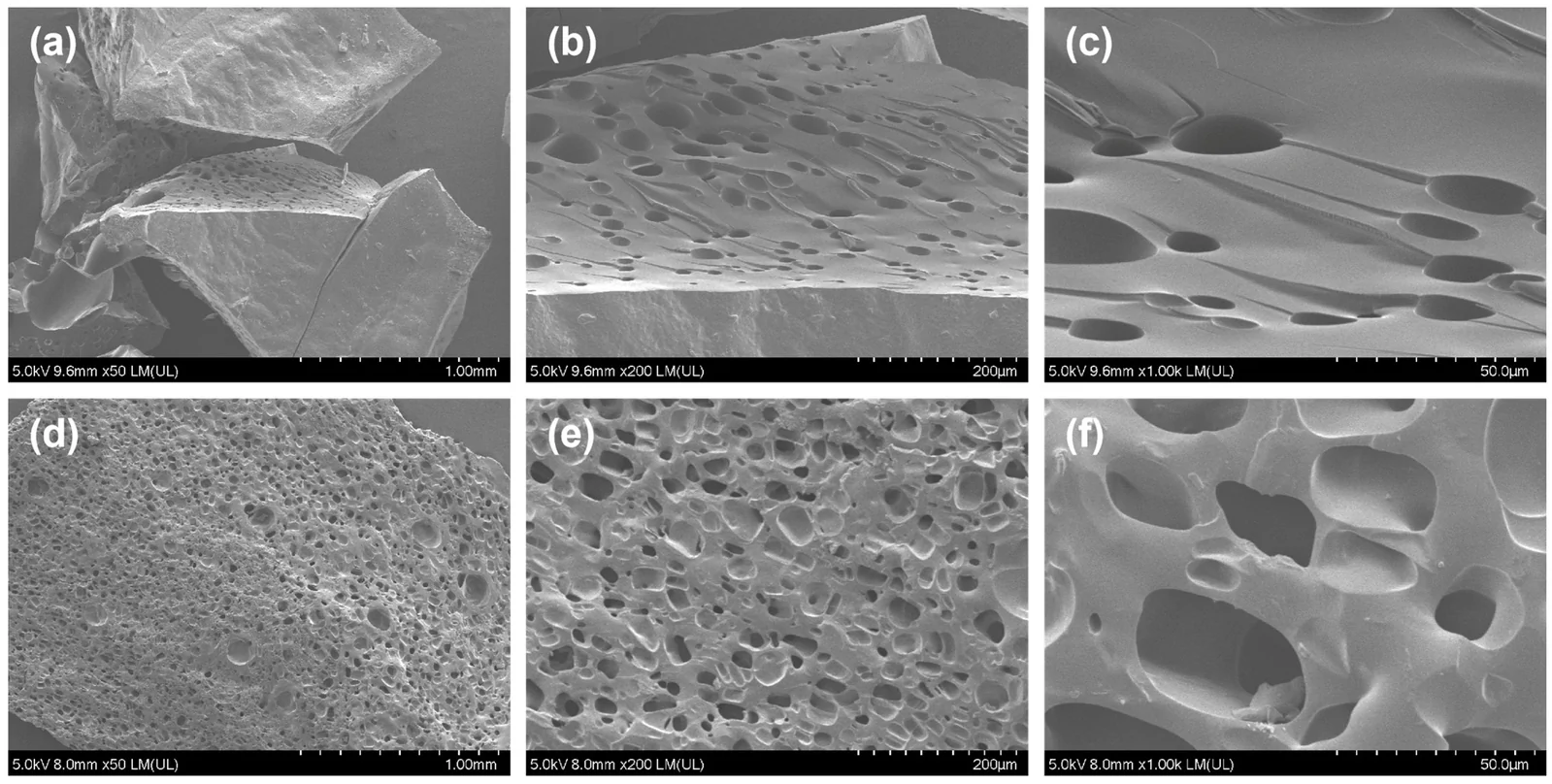
SEM images of freeze-dried samples: (**a**–**c**) P(AA-co-AM) SAP at magnifications of 50×, 200×, and 1000×; (**d**–**f**) P(AA-co-AM)/CBP SAP at magnifications of 50×, 200×, and 1000×.

The morphologies of P(AA-co-AM) SAP and P(AA-co-AM)/CBP SAP were examined by SEM at an accelerating voltage of 5.0 kV, as shown in Figure 3.

Figure 3a–c correspond to P(AA-co-AM) SAP. The structure of P(AA-co-AM) SAP is relatively compact. Although some pores are present, they are fewer in number, and the pore walls are rather smooth. This indicates that the mass-transfer channels within the pure polymer hydrogel are limited, which is unfavorable for rapid liquid penetration and diffusion during absorption.

Figure 3d–f correspond to P(AA-co-AM)/CBP SAP. In contrast to the pure SAP, the composite exhibits a visibly looser and more porous architecture, with qualitatively greater pore abundance and interconnectivity in the freeze-dried state. Moreover, the pore walls of the composite sample are relatively rough, which helps to increase the contact area between the liquid and the hydrophilic groups inside the material. It should be noted that the SEM images were obtained from as-synthesized hydrogels subjected to rapid liquid nitrogen quenching and freeze-drying. While the observed pore architecture is inevitably influenced by ice-crystal growth during the freeze-drying process, these images provide qualitative evidence that the fine CBP particles achieve good macroscopic integration with the P(AA-co-AM) matrix without obvious phase separation. Furthermore, rather than directly reflecting the true dynamic pore size in a hydrated state, this highly interconnected freeze-dried porous structure is reasonably hypothesized to potentially serve as a continuous physical transport network that facilitates the rapid diffusion of water into the interior of the hydrogel upon contact with aqueous media. This is consistent with the higher absorption capacity and faster absorption rate of the composite hydrogel [12,13,25]. Importantly, this specific surface roughness, which originates from the unrefined biomass particles, is closely analogous to the microstructural features observed in porous biopolymer hydrogels [18] and is considered a key contributor to the rapid capillary migration and molecular diffusion dynamics of the composite.

### 2.5. TGA

**Figure 4:**
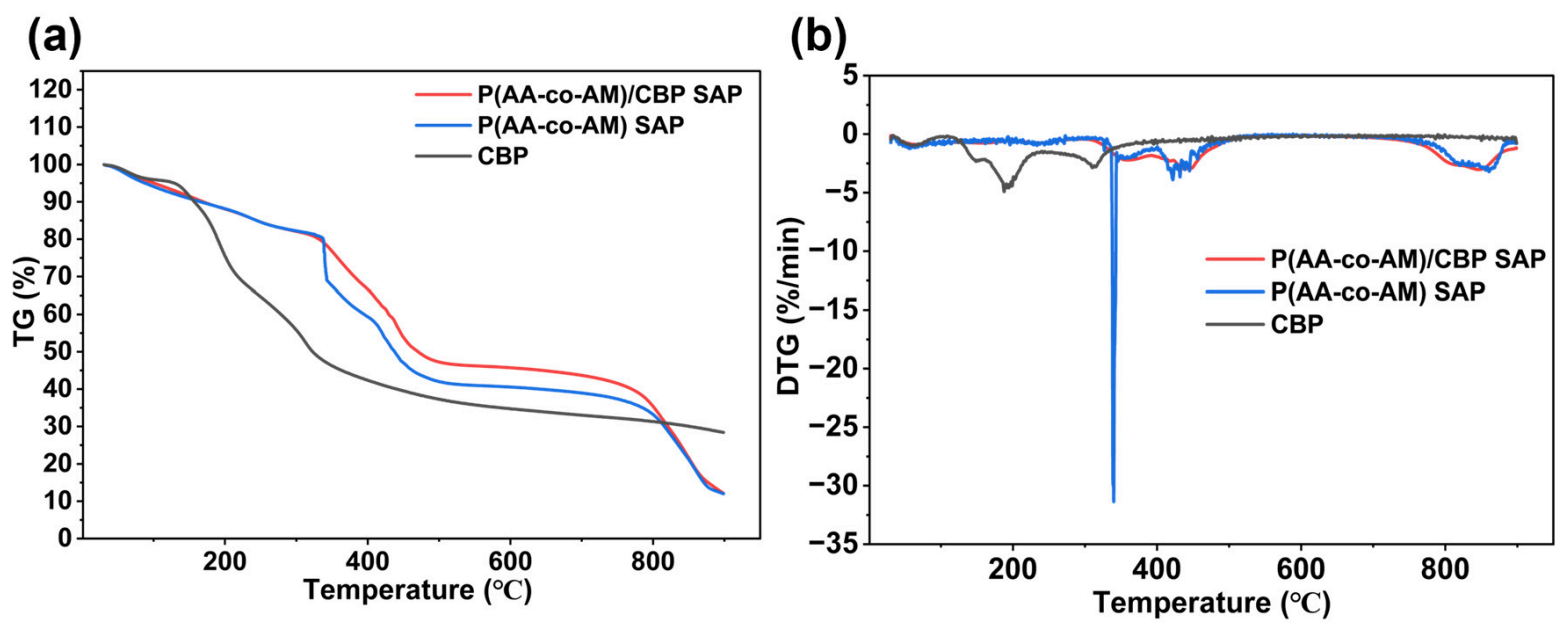
Thermal degradation profiles of CBP, P(AA-co-AM) SAP, and P(AA-co-AM)/CBP SAP: (**a**) TGA curves and (**b**) corresponding DTG curves.

The thermal decomposition behaviors of CBP, P(AA-co-AM) SAP, and P(AA-co-AM)/CBP SAP were evaluated by thermogravimetric analysis (TGA). To avoid the subjectivity of assigning boundaries to different decomposition stages and to enable a standardized scientific comparison, the original, unshifted TGA curves are presented in Figure 4. Furthermore, universal thermal degradation parameters, including $T_{5\%}$ (temperature at 5% mass loss), $T_{10\%}$ (temperature at 10% mass loss), the extrapolated onset decomposition temperature ($T_{onset}$), and the maximum degradation rate temperature ($T_{max}$), were extracted from the TGA and DTG curves and are summarized in Table 1.

All three samples exhibit multi-stage thermal decomposition behavior due to their different compositions and structures. For the unrefined CBP, the initial mass loss is primarily attributed to the evaporation of free and adsorbed water. The subsequent stages involve the stepwise degradation of biomass components, with thermally labile components (e.g., pectin and hemicellulose) decomposing first, followed by the continuous decomposition of more stable polysaccharide skeletons (e.g., cellulose) and the eventual deep carbonization of residual organic components. This stepwise degradation behavior is highly consistent with the thermal decomposition profiles of lignocellulosic biomass components reported in the literature [30].

For the pure P(AA-co-AM) SAP and the P(AA-co-AM)/CBP SAP, the initial mass loss (reflected by $T_{5\%}$ and $T_{10\%}$) is mainly associated with the evaporation of free and bound water within the hydrogel network. Following this, the hydrogels undergo a major mass loss stage, which corresponds to the large-scale random scission of the P(AA-co-AM) copolymer backbone, the thermal elimination of polar side groups (such as decarboxylation and deamination), and the concurrent degradation of the CBP-derived multicomponent biomass matrix in the composite. As clearly demonstrated by the standardized thermal parameters in Table 1, the incorporation of CBP significantly altered the thermal degradation kinetics of the polymer network. Compared with the pure P(AA-co-AM) SAP, the extrapolated onset decomposition temperature ($T_{onset}$) of the main network backbone for the P(AA-co-AM)/CBP SAP shifted substantially from 336.4 °C to 380.5 °C, while the maximum degradation rate temperature ($T_{max}$) was delayed remarkably from 339.8 °C to 438.2 °C.

This significant enhancement in thermal stability—evidenced by an increase of 44.1 °C in the $T_{onset}$ of the crosslinked network—strongly indicates the presence of intense multi-component interfacial interactions between the CBP scaffold and the polymer matrix. The multicomponent biomass matrix, combined with the extensive physical entanglement and interfacial hydrogen bonding network, effectively restricts the thermal mobility of the polymer segments at elevated temperatures, thereby significantly retarding the thermal degradation of the network framework. The subsequent mass loss observed at extremely high temperatures (>700 °C) is attributed to the deep carbonization of residual char and does not reflect the thermal stability of the original crosslinked network.

### 2.6. Optimization of Synthesis Conditions

**Figure 5:**
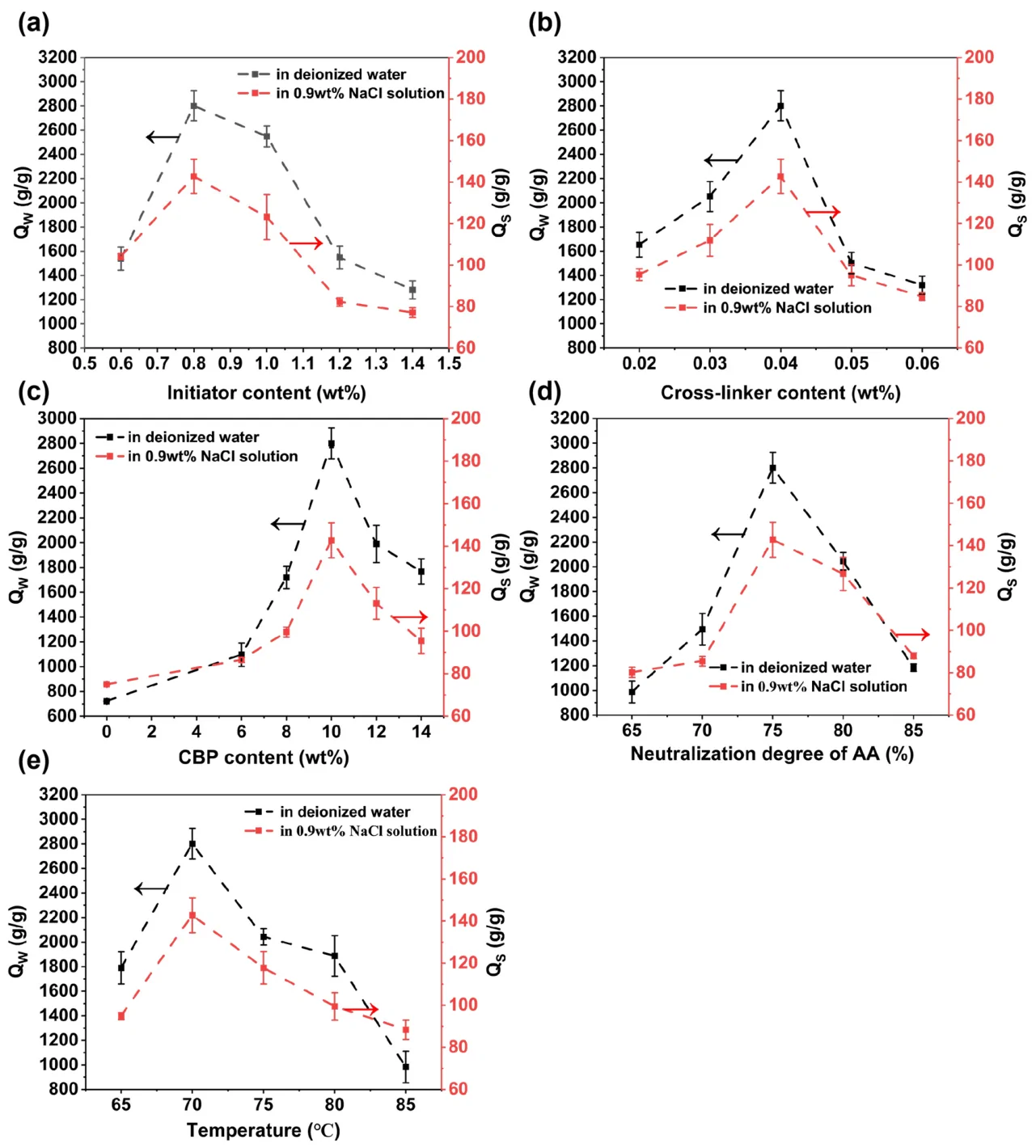
Effects of various reaction conditions on the 30 min absorption capacity of P(AA-co-AM)/CBP SAP in deionized water ($Q_{w}$) and 0.9 wt% NaCl solution ($Q_{s}$). The corresponding axes are indicated by arrows on each curve: (**a**) total initiator content (wt% relative to acrylic acid); (**b**) crosslinker (MBA) content (wt% relative to acrylic acid); (**c**) CBP content (wt% relative to acrylic acid); (**d**) neutralization degree of acrylic acid (mol% NaOH relative to AA); (**e**) reaction temperature (°C). Quantitative absorption data are presented as the mean ± standard deviation (SD) derived from three replicate measurements obtained from the same synthesized batch (*n* = 3).

To optimize the synthesis conditions, the effects of initiator content, crosslinker (MBA) content, CBP content, neutralization degree of acrylic acid, and reaction temperature on the absorption capacity of P(AA-co-AM)/CBP SAP were systematically investigated. The results are summarized in Figure 5.

As shown in Figure 5a–e, the absorption capacity of the composite SAP in both deionized water and 0.9 wt% NaCl solution exhibited a bell-shaped response to each of the five parameters. The optimal formulation was identified as: initiator content 0.8 wt%, MBA content 0.04 wt%, CBP content 10 wt%, neutralization degree 75%, and reaction temperature 70 °C. Under these conditions, the composite achieved its maximum absorption capacity in both deionized water and saline solution.

The observed trends are consistent with the classical Flory-Rehner swelling theory [31]: insufficient initiator or crosslinker leads to incomplete network formation, while excessive amounts result in over-crosslinking that restricts network expansion [32,33,34,35,36]. Specifically, an excessive amount of crosslinker significantly increases the theoretical crosslinking density, which triggers pore contraction and restricts the network space. This aligns with the trends observed in recent functional gel systems [18]. The optimal CBP content of 10 wt% reflects a balance between the reinforcing effect of the biomass scaffold and the adverse effects of excessive rigid domains on network flexibility, while overly dense grafting at the CBP surface may create localized inhomogeneities [37,38,39,40]. Similarly, the neutralization degree of 75% provides a balance where the ionized -COO^−^ groups generate sufficient electrostatic repulsion between polymer chains and osmotic driving force for network expansion, while avoiding excessive charge screening by excess Na^+^ ions present in the swelling medium that would weaken the inter-chain repulsion [14,21,41]. The reaction temperature of 70 °C balances initiator decomposition efficiency and polymerization rate, as redox polymerization kinetics are strongly temperature-dependent [42,43].

It must be pointed out that the unconstrained free-swelling and filtration method measures the maximum theoretical swelling potential of the material. Without the spatial constraints imposed by traditional tea-bag materials, the composite in this study exhibited extremely high ultimate absorption values. This is conceptually distinct from the CRC value (73.17 g/g, Table 2), which reflects the liquid retention capacity under constrained and centrifuged conditions. In future commercial translation research, it will be necessary to incorporate industry-standard methods to further evaluate its performance in actual constrained application environments.

### 2.7. Absorption and Swelling Behavior

This section systematically evaluates the absorption and swelling behavior of P(AA-co-AM)/CBP SAP, covering swelling kinetics, water retention, and cyclic swelling performance.

#### 2.7.1. Swelling Kinetics Analysis

**Figure 6:**
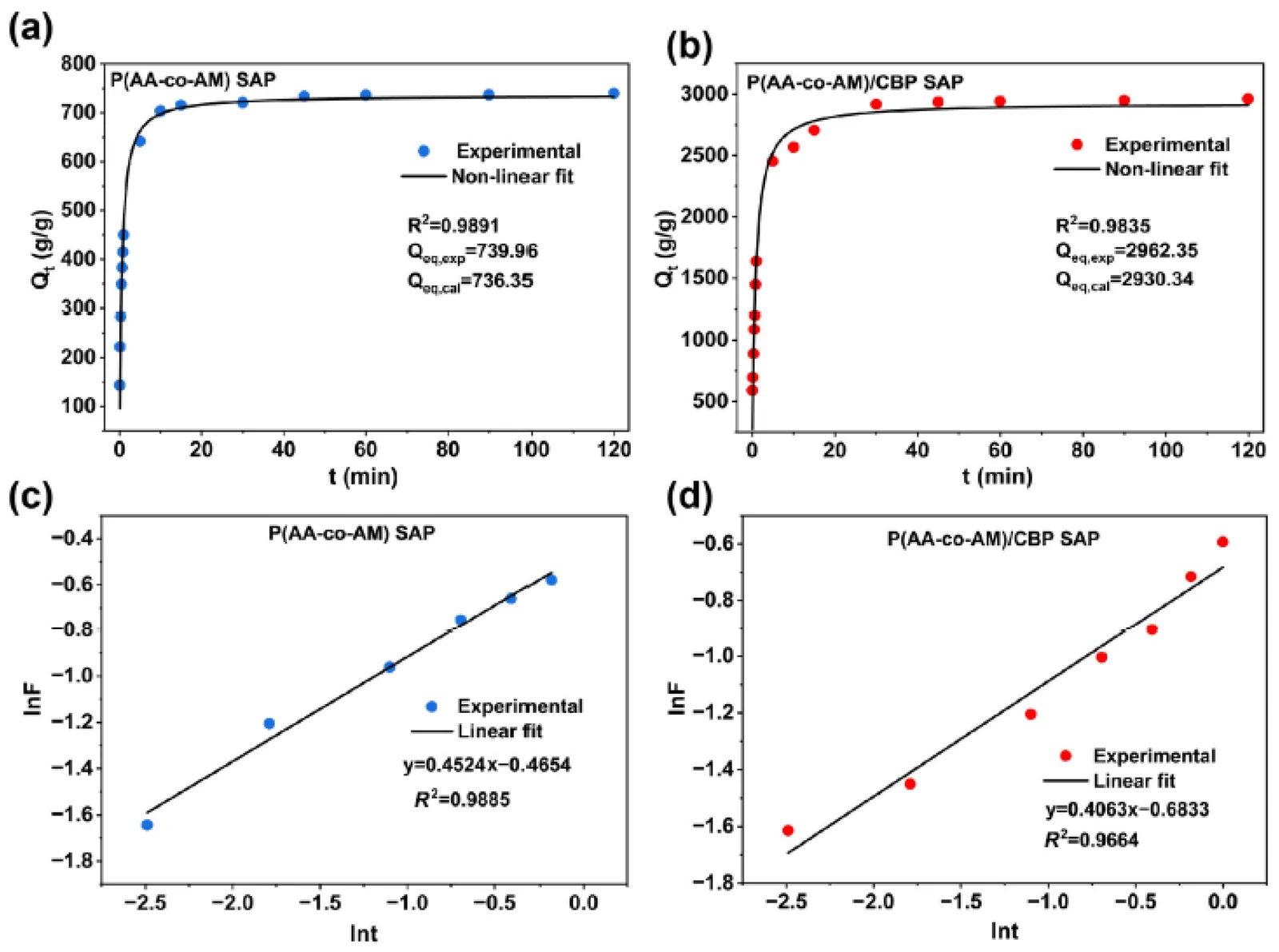
Swelling kinetics and model fitting of P(AA-co-AM) SAP and P(AA-co-AM)/CBP SAP in deionized water at 25 °C. (**a**,**b**) Experimental data ($Q_{t}$ vs. t) fitted with the non-linear pseudo-second-order kinetic model. (**c**,**d**) Linearized Fickian diffusion model fitting (ln F vs. ln t) for the initial swelling stage (F<0.6).

The swelling kinetics of P(AA-co-AM)/CBP SAP and pure P(AA-co-AM) SAP were evaluated in deionized water at room temperature, and the results are compared in Figure 6a,b. The composite achieved rapid water absorption, reaching 1637.49 g/g within 1 min and an equilibrium free-swelling absorption $Q_{eq}$ of 2962.35 g/g, significantly higher than that of the pure SAP (449.65 g/g at 1 min and 739.96 g/g at equilibrium). This enhancement is attributed to the abundant hydrophilic groups (-OH, -NH, -CONH-) provided by CBP and the interconnected porous structure revealed by SEM (Figure 3). Such porous morphology is known to facilitate liquid permeation and improve absorption performance [44].

The swelling kinetics were evaluated using the non-linear pseudo-second-order model (Figure 6a,b) to avoid the distorted error structure and inflated correlation coefficients often introduced by linearized forms. The non-linear fitting yielded $R^{2}$ values of 0.9891 for pure SAP and 0.9835 for the composite SAP. While such empirical curve fitting alone does not serve as direct mechanistic proof, the reasonable model agreement suggests that the overall swelling process is likely governed by a complex interplay of cooperative chain relaxation and water diffusion, rather than being purely diffusion-controlled [45]. The Fickian diffusion model was applied to the initial swelling stage (F<0.6), yielding diffusion exponent n values of 0.4524 for pure SAP and 0.4063 for the composite (Figure 6c,d). Both values are below 0.5, confirming pseudo-Fickian diffusion dominance in the early stage [46]. The lower n value of the composite indicates more pseudo-Fickian diffusion behavior, consistent with the perturbed chain mobility caused by the multicomponent biomass matrix and its open-pore structure [47]. Complete fitting parameters are provided in the Supplementary Information (Tables S6 and S7 and Figure S3).

#### 2.7.2. Water Retention Capability

**Figure 7:**
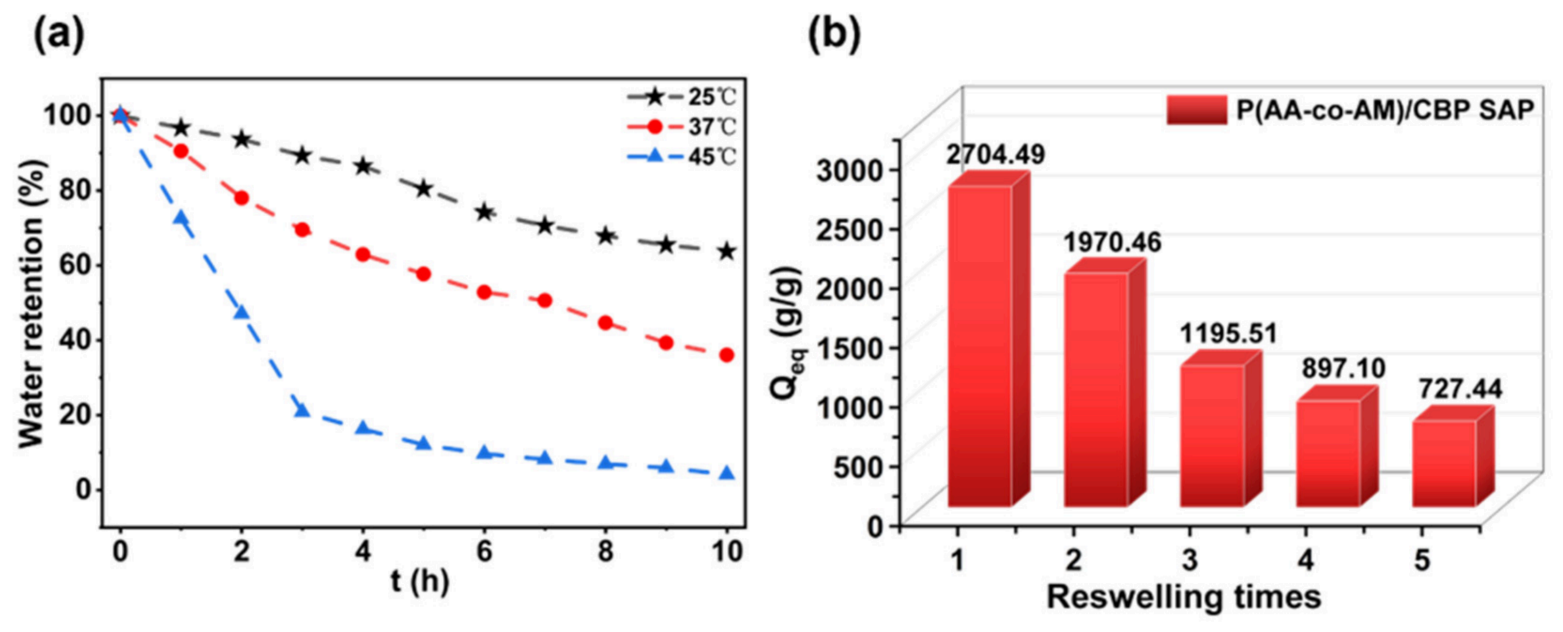
(**a**) Water retention curves of P(AA-co-AM)/CBP SAP at 25 °C, 37 °C, and 45 °C; (**b**) cyclic swelling performance of P(AA-co-AM)/CBP SAP over five swelling–drying cycles.

The water retention behavior of P(AA-co-AM)/CBP SAP was studied at 25 °C, 37 °C, and 45 °C; the results are presented in Figure 7a.

At 25 °C, the water retention rate $W_{r}$ remained at 63.72% after 10 h, indicating good water-holding capacity under ambient conditions. At 37 °C, $W_{r}$ dropped to 36.04% after 10 h, while at 45 °C, rapid water loss occurred ($W_{r}=47.08\%$ within 2 h and 4.12% after 10 h). The time required to reach 50% water retention ($t_{50}$) was >10 h at 25 °C, approximately 7.1 h at 37 °C, and approximately 1.9 h at 45 °C.

This temperature-dependent behavior is attributed to the different binding states of water within the hydrogel [48,49]. Free water in the pores is readily lost at elevated temperatures, while bound water associated with hydrophilic groups (-OH, -COO^−^, and -CONH_2_) is released more slowly. The interconnected open-pore structure, while beneficial for rapid absorption, provides low-resistance pathways for water migration upon heating. In summary, P(AA-co-AM)/CBP SAP shows adequate water retention at 25 °C and 37 °C but exhibits a sharp decline at elevated temperatures, indicating that its practical use is best limited to temperatures below 45 °C.

#### 2.7.3. Cyclic Swelling Behavior

The cyclic absorption performance of P(AA-co-AM)/CBP SAP was evaluated over five swelling–drying cycles, and the results are shown in Figure 7b.

As shown in Figure 7b, the cyclic absorption capacity of P(AA-co-AM)/CBP SAP decreases non-linearly with the number of cycles. The highest equilibrium absorption is observed in the first cycle (2704.49 g/g). In the second cycle, the absorption capacity decreases to 1970.46 g/g, which is approximately 72.86% of the first-cycle value. As cycling continues, the decline gradually slows, and a residual absorption capacity of approximately 26.90% of the initial value is still maintained after five cycles. It should be noted that the absorption capacity in the first cycle (2704.49 g/g) is lower than the equilibrium capacity obtained in the swelling kinetics test (2962.35 g/g). This difference arises from the distinct liquid volumes used in the experimental protocols: to facilitate repeated complete recovery and drying in standard glassware, the cyclic test utilized a restricted liquid volume of 300 mL. Because 0.1000 g of the highly absorbent composite rapidly depletes the free water in a 300 mL system, the concentration gradient and terminal osmotic driving force drop significantly toward the end of the swelling period, resulting in somewhat lower ultimate swelling compared to the 500 mL system used for the kinetics evaluation.

This decay can be attributed to irreversible structural changes during the cycles. During the high-temperature drying stage, the gel undergoes volume shrinkage, and the gel network experiences partial structural collapse or local densification. As a result, some of the effective mass-transfer channels are reduced or lost, limiting the expandable space in subsequent swelling [50]. At the same time, repeated soaking may cause the loss of weakly bound or slightly soluble components, which can alter the hydrophilic sites and the microstructure of the pore walls, further reducing the absorption capacity [50]. For practical application scenarios requiring an extended reuse lifespan, alternative milder drying methods (such as low-temperature vacuum drying or fluid-bed drying) are strongly recommended to better preserve the interfacial hydrogen bonding networks and mitigate structural collapse.

Despite the significant decrease in cyclic absorption capacity, the result of 727.44 g/g after five drying–swelling cycles indicates that the composite exhibits a partial residual swelling capacity.

### 2.8. Mechanical Properties and Viscoelastic Behavior

The mechanical properties of P(AA-co-AM)/CBP SAP were evaluated through rheological analysis and AUL. Rheological characterization provides insights into the network stiffness, elastic dominance, and viscoelastic stability, while AUL reflects the absorption capacity under external pressure. The results are presented in the following subsections.

#### 2.8.1. Rheological Analysis

**Figure 8:**
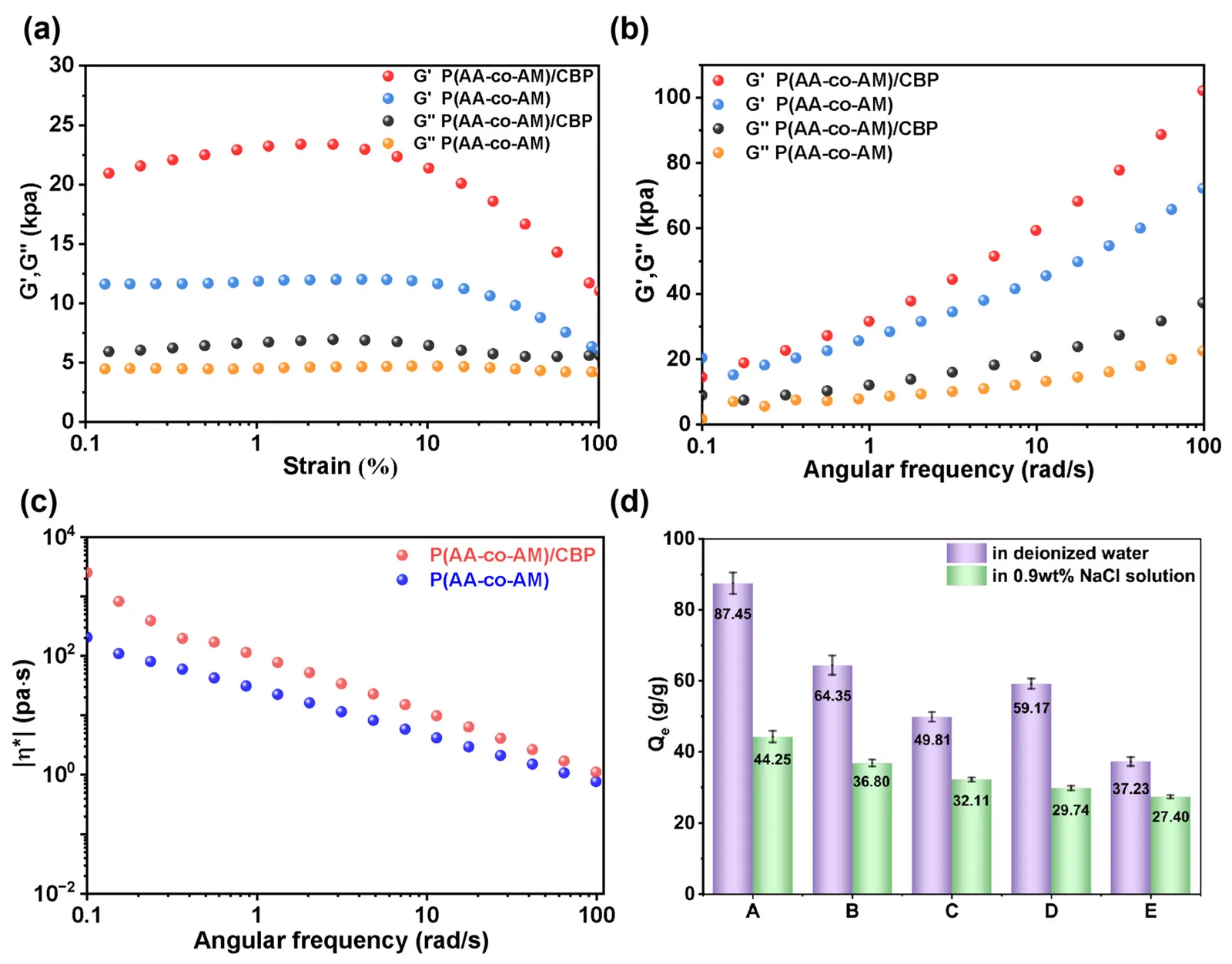
(**a**–**c**) Rheological properties of P(AA-co-AM) SAP and P(AA-co-AM)/CBP SAP: (**a**) strain sweep curves showing the storage modulus (G′) and loss modulus (G″) as functions of strain amplitude; (**b**) dynamic frequency sweep curves showing G′ and G″ as functions of angular frequency; (**c**) complex viscosity (|η*|) as a function of angular frequency. All rheological measurements were performed at 25 °C. The frequency sweeps were conducted at a constant strain of 0.05% within the low-strain regime (well below the peak strain). (**d**) AUL values of P(AA-co-AM)/CBP SAP (A), pure P(AA-co-AM) SAP (B), and three representative commercial SAPs (C: SAP1, D: SAP4, and E: SAP6) in deionized water and 0.9 wt% NaCl solution. All commercial SAP samples were tested as received without secondary grinding or physical sieving. The AUL data for the synthesized SAPs (A and B) were derived from three replicate measurements obtained from the same synthesized batch (*n* = 3), while data for the commercial products were obtained from three independent measurements (*n* = 3). The error bars represent the standard deviation (SD). Prior to testing, all commercial SAP samples were strictly stored in sealed containers at room temperature.

Rheological characterization is a well-established method for evaluating the crosslinked network structure and viscoelastic response of hydrogels [51,52]. The storage modulus (G′) reflects the elastic energy stored during deformation, representing the network strength, while the loss modulus (G″) reflects the viscous dissipation behavior associated with chain motion and internal friction. To investigate the effect of CBP incorporation on the network structure and mechanical stability of the P(AA-co-AM) hydrogel, dynamic frequency sweep measurements were performed on pure P(AA-co-AM) SAP and P(AA-co-AM)/CBP SAP. The results are presented in Figure 8a–c.

As shown in Figure 8a, the G′ of both P(AA-co-AM) SAP and P(AA-co-AM)/CBP SAP, as well as the G″ of the composite, initially increased with increasing strain, reaching a maximum at approximately 2.43%, 3.64%, and 2.81% strain, respectively, before decreasing at higher strains. In contrast, the G″ of the pure P(AA-co-AM) SAP did not show a clear initial increase and remained relatively stable before decreasing. A classical linear viscoelastic region (LVR) with a constant G′ was not observed for either hydrogels; instead, the initial increase in moduli suggests the presence of strain-induced structural rearrangements or weak associations (e.g., hydrogen bonds) within the network. This behavior is commonly observed in physically crosslinked or hydrogen-bonded hydrogels, where reversible non-covalent interactions provide structural adaptability under small deformations [53]. The initial increase may therefore reflect the alignment of polymer chains and/or the reformation of hydrogen bonds under small deformations, which temporarily enhances the network stiffness.

Beyond the maximum, G′ decreased gradually. Notably, the P(AA-co-AM)/CBP SAP exhibited a faster decrease in G′ at high strains compared with the pure P(AA-co-AM) SAP, starting from a higher maximum value and declining more rapidly. This indicates that the CBP-reinforced network, while initially stiffer and stronger, is more susceptible to strain-induced disruption once the deformation exceeds a critical threshold—a typical feature of rigidized networks. Nevertheless, despite this faster decline, the G′ values of the P(AA-co-AM)/CBP SAP remained consistently higher than those of the pure P(AA-co-AM) SAP across the entire strain range (Figure 8a), confirming that the CBP-reinforced network maintains superior stiffness even at large deformations. Measurements were performed on an Anton Paar MCR302 rheometer, which has a minimum detectable torque limit of 0.05 μN·m. During our measurements, the actual generated torque ranged from 0.1 to 5.0 μN·m, which is strictly more than twice the lower detection limit. Furthermore, an empty-load baseline test was conducted under identical conditions; the baseline modulus was more than an order of magnitude lower than the sample signals, yielding a signal-to-noise ratio (SNR) strictly greater than 10. These instrument validation metrics confirm that the observed initial rise in modulus is a genuine material response that may reflect structural rearrangements, rather than being masked by low-torque limitations or instrument noise. This behavior underscores a key trade-off in rigidized networks: an earlier onset of strain-induced softening and faster post-peak softening, yet the absolute mechanical performance remains superior to that of the less rigid counterpart. The maximum G′ values were 23.4 kPa for the composite vs. 11.9 kPa for the pure SAP, confirming the overall reinforcing effect of CBP. This interfacial hydrogen bonding network, constructed by multiple components such as hydroxyl and amide groups within CBP, exhibits a structural reinforcement effect analogous to the coordinate hydrogen and ionic bonding networks observed in refined biopolymers (e.g., chitosan–alginate–carrageenan blends) [54].

To ensure minimal network disturbance for subsequent frequency sweep measurements, a constant strain of 0.05% (within the initial low-strain regime, well below the peak strain) was selected. This strain is sufficiently small to preserve the integrity of the network while maintaining an adequate signal-to-noise ratio.

Figure 8b presents the dynamic frequency sweep results, obtained at a constant strain of 0.05% and 25 °C. Over the entire frequency range (0.1–100 rad s^−1^), G′ remained higher than G″ for both samples, with no crossover point, confirming that both hydrogels maintain a gel state structure with elastic dominance. The G′ values of the P(AA-co-AM)/CBP SAP were consistently higher than those of the pure P(AA-co-AM) SAP across the entire frequency range and increased more rapidly with frequency, indicating that the introduction of CBP constructs a more rigid network with enhanced chain constraints. This enhancement primarily reflects the substantial physical reinforcement provided by the incorporated multicomponent biomass matrix and extensive interfacial hydrogen bonding, rather than an increase in absolute chemical crosslinking density. The loss modulus (G″) of both samples was much lower than G, and showed even weaker frequency dependence than G′, which is consistent with the behavior expected for chemically crosslinked hydrogels, where the elastic response dominates over viscous dissipation.

The loss factor (tanδ = G″/G′) for the P(AA-co-AM)/CBP SAP was 0.26, lower than that of the pure P(AA-co-AM) SAP (0.36). This decrease in tanδ indicates a more elastic and less dissipative network, with improved structural stability and recovery capability under dynamic deformation.

As shown in Figure 8c, both hydrogels exhibited a decrease in complex viscosity (|η*|) with increasing angular frequency, a typical shear-thinning behavior. The P(AA-co-AM)/CBP SAP showed significantly higher |η*| values than the pure P(AA-co-AM) SAP across the entire frequency range. This indicates that the particulate reinforcement from the multicomponent biomass matrix effectively restricts polymer chain mobility, resulting in a higher overall viscosity level without necessitating a higher degree of covalent crosslinking.

Overall, the rheological analysis confirms that the incorporation of CBP enhances the network stiffness, elastic dominance, and viscoelastic stability of the P(AA-co-AM) hydrogel, albeit with a more strain-sensitive response at high deformations—a typical trade-off for rigidized networks. These rheological findings provide direct evidence of interfacial reinforcement arising from hydrogen bonding between the CBP surface and the polymer network, highlighting the role of bio-based interfaces in modulating the colloidal network structure of the hydrogel. These findings are consistent with the improved thermal stability (Section 2.5) and will be further validated by AUL performance in the following subsection.

While these results clearly demonstrate the magnitude of the CBP-induced reinforcing effect, it should be noted that only upward strain sweeps were performed in this proof-of-concept study. The evaluation of hysteresis via downward sweeps, along with inferential statistical comparisons across multiple independent batches, remains an area for future fundamental rheological investigations.

#### 2.8.2. AUL Performance

The AUL of P(AA-co-AM)/CBP SAP, pure P(AA-co-AM) SAP, and the commercial SAPs was measured, and the results are shown in Figure 8d. AUL is a key performance indicator for evaluating superabsorbent materials under practical application conditions [55].

In deionized water, the AUL value of P(AA-co-AM)/CBP SAP reached 87.45 g/g, significantly higher than those of pure P(AA-co-AM) SAP (64.35 g/g) and the three representative commercial products (SAP1: 49.81 g/g, SAP4: 59.17 g/g, and SAP6: 37.23 g/g). This improvement is primarily attributed to the multicomponent biomass matrix of CBP, which acts as a physical reinforcement within the P(AA-co-AM) network. The abundant hydroxyl groups on CBP can form hydrogen bonds with the polymer chains, further stabilizing the network structure. Under external load, the reinforced network resists pore collapse and network contraction, maintaining continuous liquid diffusion channels and thereby preserving a high absorption capacity even under pressure. A similar reinforcing effect has been reported for cellulosic fillers in superabsorbent hydrogels [37,56].

In 0.9 wt% NaCl solution, the AUL values of all samples were considerably lower than those in deionized water. P(AA-co-AM)/CBP SAP and pure P(AA-co-AM) SAP exhibited AUL values of 44.25 and 36.80 g/g, respectively, while the three commercial products (SAP1, SAP4, and SAP6) showed values of 32.11, 29.74, and 27.40 g/g, respectively. The lower AUL in saline solution is mainly due to charge screening by Na^+^ ions, which weakens the electrostatic repulsion among -COO^−^ groups on the polymer backbone and reduces the Donnan osmotic pressure difference [14,57]. This screening effect also reduces the effective water affinity of the polymer network. Under an external load, the already weakened polymer network is more susceptible to collapse, leading to pore closure and restricted liquid diffusion, which further reduces the absorption capacity under load compared with free-swelling conditions.

The superior AUL performance of P(AA-co-AM)/CBP SAP in saline solution, despite these adverse effects, can be attributed to two key features of the CBP reinforcement. First, the multicomponent biomass matrix of CBP acts as a physical scaffold that helps preserve pore channels against collapse under external load, even in a saline environment. Second, the abundant hydrophilic groups on the CBP surface provide additional water-binding sites, partially compensating for the reduced water affinity caused by charge screening. These structural roles are supported by the SEM images (Figure 3), which reveal a well-developed interconnected porous architecture that facilitates rapid liquid entry and maintains continuous diffusion channels. Consequently, P(AA-co-AM)/CBP SAP exhibits superior AUL performance even under saline conditions.

Thus, the incorporation of cucumber biomass powder significantly enhances the AUL performance of P(AA-co-AM)/CBP SAP. In both deionized water and saline solution, within this confined dialysis-membrane microenvironment characterized by specific mass-transfer resistance, the composite exhibits an absorption potential superior to that of the tested commercial products. These results confirm that CBP plays a dual role: its multicomponent biomass matrix reinforces the structural stability and pressure resistance of the hydrogel network, while its abundant hydrophilic groups help to maintain water affinity even in saline environments. The enhanced AUL performance, together with the rheological evidence of network stiffening (Section 2.8.1), collectively demonstrates that the interfacial hydrogen bonding and rigid scaffold provided by CBP effectively improve the mechanical stability of the hydrogel under load.

### 2.9. Performance Comparison with Commercial SAPs

The absorption performance of P(AA-co-AM)/CBP SAP was compared with that of the control sample (P(AA-co-AM) SAP without CBP) and eleven commercial SAPs (SAP1-SAP11), including leading imported brands widely used in commercial absorbent products in the Chinese market. Similar comparative assessments of commercial SAPs have been reported using standardized protocols such as FSC, CRC, and AUL measurements [58]. The results of our comparison are presented in Table 2.

The commercial SAPs (SAP1-SAP11) include leading imported brands widely used in commercial absorbent products. AUL values reported for the representative commercial SAPs (SAP1, SAP4, and SAP6) were individually obtained from three independent measurements (*n* = 3). Detailed product information is provided in the Supplementary Information (Tables S4 and S5).

Consistent with typical polyelectrolyte hydrogels, the swelling behavior of the P(AA-co-AM)/CBP SAP exhibits pronounced salt sensitivity [59,60]. When the external medium is changed from deionized water to 0.9 wt% NaCl solution or to the more complex synthetic urine, the absorption capacity of the material shows varying degrees of reduction. The centrifuge retention capacity (CRC), measured in 0.9 wt% NaCl solution, is also included in Table 2 for comparison. This salt sensitivity arises from two main factors. On the one hand, the presence of cations (e.g., Na^+^) in the external solution introduces additional mobile ions that penetrate the hydrogel network, leading to electrostatic shielding of the fixed -COO^−^ charges on the polymer backbone, which significantly weakens the inter-chain electrostatic repulsion [14]. On the other hand, the reduction in the ion concentration gradient between the internal and external solutions decreases the Donnan osmotic pressure driving force, thereby limiting further network expansion [13]. Particularly in the synthetic urine system, in addition to the charge screening effect of monovalent inorganic ions, the presence of small-molecule nitrogen-containing organic compounds such as urea and creatinine introduces competitive hydrogen bonding interactions with the -COOH and -CONH_2_ groups of the network. These interactions reduce the number of available binding sites for water molecules and weaken the hydration of the polymer chains, thereby limiting network expansion and resulting in a more pronounced decrease in the absorption capacity compared with that in simple saline solution.

As shown in Table 2, the unconstrained free-swelling capacities of P(AA-co-AM)/CBP SAP within 30 min in deionized water, 0.9 wt% NaCl solution, and synthetic urine are 2801.00, 142.72, and 65.04 g/g, respectively, with a CRC of 73.17 g/g. Compared with the control P(AA-co-AM) SAP, the absorption capacities of P(AA-co-AM)/CBP SAP in deionized water, saline solution, and synthetic urine increase by approximately 3.9-fold, 1.9-fold, and 1.5-fold, respectively, while the CRC increases by approximately 1.6-fold. These results indicate that the introduction of CBP not only significantly enhances the swelling ability in deionized water and saline solution but also maintains a high absorption level in synthetic urine (which more closely mimics real excretion fluids) and good liquid-retention capability.

A further comparison with the eleven commercial SAPs shows that the absorption capacities of the commercial samples in synthetic urine mainly range from 34.41 to 51.87 g/g, with CRC values mostly between 30.07 and 37.87 g/g. In contrast, P(AA-co-AM)/CBP SAP exhibits a synthetic urine absorption capacity of 65.04 g/g and a CRC of 73.17 g/g, both of which are significantly higher than the corresponding ranges of the commercial samples. Nevertheless, P(AA-co-AM)/CBP SAP still exhibits superior combined absorption-retention performance compared with both the control and the commercial SAPs. These results demonstrate that P(AA-co-AM)/CBP SAP possesses excellent absorption and liquid-retention performance in saline and synthetic urine environments, underscoring the effectiveness of interfacial hydrogen bonding in maintaining appreciable absorption potential and structural stability in saline environments for colloidal hydrogel systems.

## 3. Conclusions

A high-performance composite superabsorbent polymer was successfully constructed by incorporating unrefined cucumber biomass powder (CBP) as a renewable reinforcing scaffold into a P(AA-co-AM) network via free-radical polymerization. Spectroscopic and diffraction analyses indicate that the multicomponent biomass matrix is effectively integrated into the polymer network. This integration is consistent with extensive interfacial hydrogen bonding and potential partial chemical integration, rather than solely relying on simple physical blending. In the freeze-dried state, the composite exhibited a more open and interconnected porous architecture, as qualitatively suggested by SEM, and simultaneously enhanced its thermal stability, raising the standardized extrapolated onset decomposition temperature ($T_{onset}$) from 336.4 °C to 380.5 °C.

Under optimized synthesis conditions (initiator 0.8 wt%, MBA 0.04 wt%, CBP 10 wt%, neutralization degree 75%, 70 °C), the composite exhibited exceptional 30 min free-swelling absorption capacities of 2801.00 g/g in deionized water, 142.72 g/g in 0.9 wt% NaCl, and 65.04 g/g in synthetic urine, with a centrifuge retention capacity of 73.17 g/g in 0.9 wt% NaCl. The material also demonstrated rapid swelling kinetics (1637.49 g/g within 1 min), a partial residual swelling capacity (727.44 g/g retained after five cycles), and a clear temperature-dependent water loss behavior. Notably, rheological analysis and absorption-under-load measurements demonstrated that the multicomponent biomass matrix not only reinforces the network structure but also improves its viscoelastic stability and pressure resistance, albeit at the cost of increased strain sensitivity. This work demonstrates that unrefined model biomass can serve as an effective, low-cost scaffold for functional polymer composites, offering a sustainable pathway toward bio-based absorbent materials. While cucumber powder was used as a readily available model system to validate this proof-of-concept strategy, the key methodological insight—that crude, unprocessed biomass can be directly integrated into a polymer network without prior purification—may inform the design of bio-based SAPs from a broader range of renewable feedstocks.

It should be noted that the direct use of natural unrefined biomass faces challenges of compositional complexity and heterogeneity. While a homogenized sample was used here for proof-of-concept, large-scale batch variability resulting from different geographic origins or growing seasons remains to be evaluated. Furthermore, given the inherent presence of proteins and soluble sugars in the unrefined CBP and the absence of residual monomer (e.g., acrylamide), cytotoxicity, and extractables testing in this study, any reference to personal care applications represents only a potential future direction requiring rigorous purification and comprehensive pre-clinical evaluation.

## 4. Materials and Methods

### 4.1. Materials

Fresh cucumber was obtained from a local market in Guangzhou, Guangdong Province, China. Acrylic acid (AA, chemically pure), acrylamide (AM, analytical grade), sodium hydroxide (NaOH, analytical grade), N,N′-methylenebisacrylamide (MBA, analytical grade), sodium bisulfite (NaHSO_3_, analytical grade), and anhydrous ethanol (analytical grade) were purchased from Tianjin Damao Chemical Reagent Factory (Tianjin, China). Ammonium persulfate (APS, analytical grade) and activated carbon were acquired from Sinopharm Chemical Reagent Co., Ltd. (Shanghai, China). Sodium chloride (NaCl, analytical grade) was obtained from Guangdong Chemical Reagent Engineering Technology Research and Development Center (Guangzhou, China). Synthetic urine (model PH1879, pH = 5.7, containing creatinine) was purchased from Fuzhou Feijing Biotechnology Co., Ltd. (Fuzhou, China). Deionized water was used throughout the experiments.

### 4.2. Preparation of Cucumber Biomass Powder (CBP)

Fresh cucumbers were peeled, cored, and sliced. These specific pretreatment steps were performed because the cucumber peel is rich in hydrophobic cuticular wax, while the seeds contain a high proportion of plant lipids. Removing these highly hydrophobic components is crucial to prevent severe microscopic phase separation during the subsequent aqueous free-radical polymerization, thereby ensuring good interfacial compatibility between the hydrophilic monomers and the multicomponent biomass matrix. The slices were air-dried for three days and then further dried in a vacuum oven at 75 °C to remove residual moisture. The dried cucumber slices were pulverized using a grinder. The resulting powder was designated as CBP and stored in a sealed container.

### 4.3. Preparation of P(AA-co-AM)/CBP SAP

**Figure 9:**
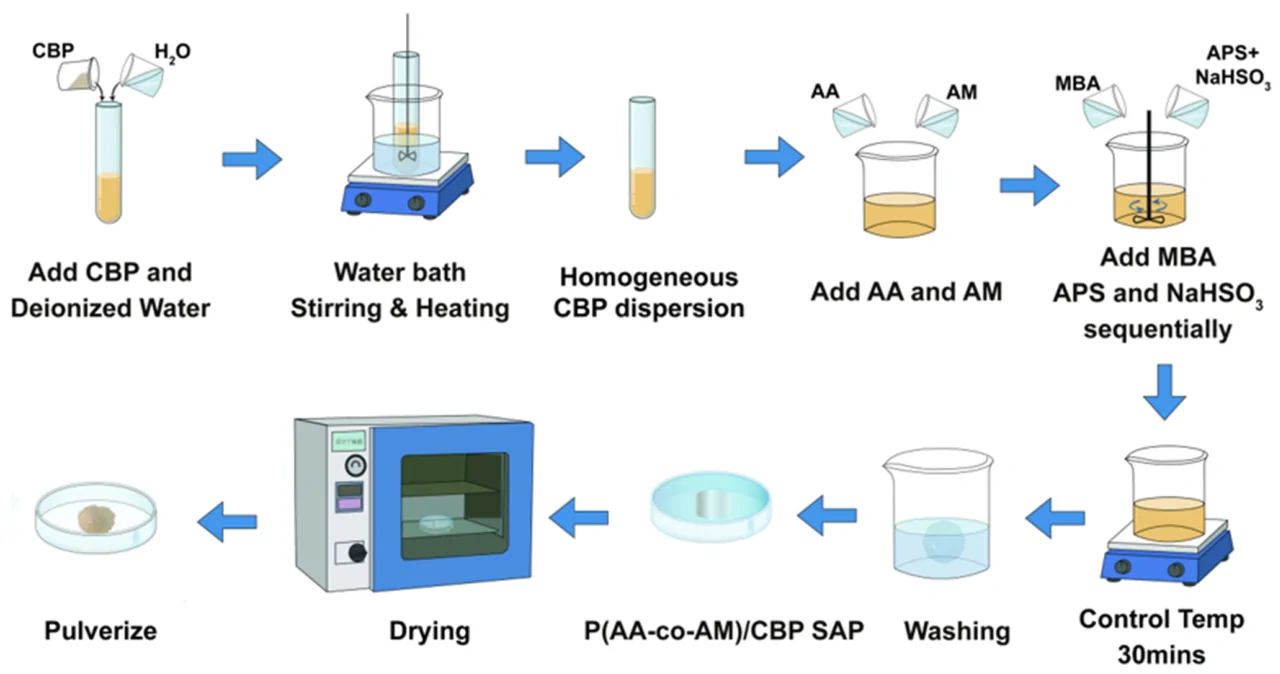
Schematic diagram of the preparation process for P(AA-co-AM)/CBP SAP.

CBP at 10 wt% (relative to the mass of acrylic acid (AA)) and deionized water were placed in a 50 mL glass tube and pre-dispersed under magnetic stirring in a 70 °C water bath for 30 min to allow full hydration of the CBP components. Partially neutralized acrylic acid and acrylamide solutions were then added, followed by the crosslinker (MBA) solution under stirring. Subsequently, APS and NaHSO_3_ solutions were added dropwise sequentially into the system within approximately 1 min to initiate the graft copolymerization. The reaction was continued at the same temperature for 30 min to form the three-dimensional network. After the reaction, the product was cooled, taken out, cut into pieces, washed with anhydrous ethanol and filtered to remove unreacted monomers and soluble components. Specifically, the gel pieces were immersed in approximately 30 mL of anhydrous ethanol and washed continuously under mild shaking for 3 cycles (each cycle lasting 30 min), followed by transfer to an oven at 75 °C for drying to constant weight. The dried product was ground and sieved through a 40-mesh screen to obtain the P(AA-co-AM)/CBP SAP. Detailed formulation parameters are provided in Table S1 in the Supporting Information. For comparison, a control sample (P(AA-co-AM) SAP) was prepared under the same conditions without adding CBP. The preparation procedure is illustrated in Figure 9.

### 4.4. Physicochemical Characterization

FTIR, XRD, SEM, and TGA were used to characterize the physicochemical properties of the samples.

Fourier transform infrared (FTIR) spectra were recorded on a Nicolet iS50 spectrometer (Thermo Fisher Scientific, Waltham, MA, USA) using the KBr pellet method. All spectra were collected with air as the background. The scanning range was 4000–400 cm^−1^ for CBP, P(AA-co-AM) SAP, and P(AA-co-AM)/CBP SAP, and 4000–500 cm^−1^ for the physical mixture.

X-ray diffraction (XRD) patterns were obtained on a BRUKER D8 ADVANCE diffractometer (Bruker Corporation, Billerica, MA, USA) over a 2θ range of 5–90° at a scan rate of 2°/min. The samples measured were CBP, P(AA-co-AM) SAP, and P(AA-co-AM)/CBP SAP.

The morphology and pore structure of the samples were observed using an SU8100 field-emission scanning electron microscope (Hitachi High-Tech Corporation, Tokyo, Japan). Before imaging, the as-synthesized hydrogels (without undergoing any drying or re-swelling processes) were directly cut into pieces, rapidly quenched in liquid nitrogen, and freeze-dried. The dried samples were mounted on conductive tape, sputter-coated with gold to enhance conductivity, and observed at an accelerating voltage of 5.0 kV.

Thermogravimetric analysis (TGA) was performed on an NETZSCH TG 309 Libra thermogravimetric analyzer (NETZSCH-Gerätebau GmbH, Selb, Germany). Approximately 5–10 mg of each sample was heated from 30 °C to 900 °C at a heating rate of 10 °C/min under a nitrogen atmosphere. The samples tested were CBP, P(AA-co-AM) SAP, and P(AA-co-AM)/CBP SAP.

### 4.5. Absorption and Liquid-Retention Performance

The absorption and liquid-retention performance of the SAP samples was evaluated using the following procedures.

#### 4.5.1. Absorption Capacity

The absorption capacity was measured using a free-swelling and filtration method in deionized water, 0.9 wt% NaCl solution, and synthetic urine at a test temperature of 25 °C. In a typical measurement, a precisely weighed dry sample mass of 0.1000 g was placed into a beaker containing an excess liquid volume of 1000 mL and allowed to swell freely for 30 min without physical restraint. Afterward, the swollen hydrogel mixture was poured onto a 100-mesh stainless steel sieve and allowed to drain freely for exactly 15 min until no continuous dripping was observed. The residual surface water was carefully wiped from the bottom of the sieve before final weighing [56]. The absorption capacity was calculated according to Equation (1):

(1) $$Q_{30min}=(M_{1}-M_{0})/M_{0}$$

where $Q_{30min}$ is the absorption capacity of SAP within 30 min (g/g), $M_{0}$ is the initial dry weight of the sample (g), and $M_{1}$ is the weight after absorption and draining (g).

#### 4.5.2. Swelling Kinetics

The swelling kinetics were studied by immersing 0.1000 g of dry SAPs in 500 mL of deionized water at room temperature. At predetermined time intervals, each sample was taken out, drained of free surface liquid using a sieve, and weighed. The absorption capacity at time *t*, $Q_{t}$, was calculated using Equation (1). The equilibrium absorption capacity $Q_{eq}$ was taken as the value at 120 min, where no further significant change in mass was observed. The experimental data were fitted to the non-linear pseudo-second-order kinetic model (Equation (2)) to avoid the error-structure distortion and inflated correlation coefficients often associated with linearized fitting [61]. Complete fitting parameters for both models are provided in the Supplementary Information (Tables S6 and S7 and Figure S3).

(2) $$Q_{t}=\frac{kQ_{eq}^{2}t}{1+kQ_{eq}t}$$

where k is the rate constant of the non-linear pseudo-second-order swelling kinetics. Furthermore, to elucidate the initial water transport mechanism, the complete Fickian diffusion model was applied to the early swelling stage (F<0.6), described by Equation (3) [62]:

(3) $$F=\frac{Q_{t}}{Q_{eq}}=k\prime t^{n}$$

which can be linearized as $\ln\;F=\;\ln\;k^{\prime }+n\;\ln\;t$. Here, F is the fractional absorption at time t, $k^{\prime }$ is a structural characteristic constant of the polymer network, and n is the characteristic diffusional exponent. Given that the dry SAPs were mechanically pulverized, the specimen geometry in this study was assumed to consist of irregular micro-particles. According to classical diffusion theory for particulate geometries, ideal Fickian diffusion is typically characterized by an exponent close to 0.43–0.5. Therefore, calculated n values of less than 0.5 were classified as pseudo-Fickian (or less-Fickian) diffusion, indicating that the water penetration is highly concentration-dependent and partially restricted by the relaxation time of the macromolecular network.

#### 4.5.3. Water Retention

Fully swollen P(AA-co-AM)/CBP SAP samples (50 g each) were placed in beakers and stored in constant-temperature incubators at 25 °C, 37 °C, and 45 °C. The weight of the samples was checked at regular intervals, and the water retention rate was calculated relative to the initial mass of absorbed water to accurately represent water loss, according to Equation (4):

(4) $$W_{r}\left(\%\right)=\frac{W_{i}-W_{dry}}{W_{0}-W_{dry}}\times 100\%$$

where $W_{r}$ is the water retention rate (%), $W_{i}$ is the mass of the hydrogel after a certain time at a given temperature, $W_{0}$ is the initial total mass of the fully swollen hydrogel (50 g), and $W_{dry}$ is the mass of the dry polymer within the sample. Given the extremely high equilibrium swelling capacity of the composite (2962.35 g/g), the dry polymer mass $W_{dry}$ in a 50 g swollen sample is exceptionally small (approximately 0.0169 g). For this qualitative analysis experiment, its impact is mathematically negligible and does not affect the overall water loss trend.

#### 4.5.4. Cyclic Swelling Test

Cyclic swelling tests were performed by repeatedly swelling and drying the same SAP sample [63]. In each cycle, 0.1000 g of dried SAP was immersed in 300 mL of deionized water for 2 h to reach equilibrium swelling. The swollen hydrogel was then dried in a forced-air oven at 105 °C to constant weight and used in the next cycle. The absorption capacity in each cycle was calculated using Equation (1).

#### 4.5.5. Centrifuge Retention Capacity (CRC)

CRC was measured according to ISO 17190-6:2020 [64]. A dry sample (0.1000 g) was swollen in 0.9 wt% NaCl solution for 30 min, drained for 10 min, and then centrifuged at 1460 rpm for 3 min. The weight of the gel after centrifugation was recorded, and CRC was calculated using Equation (5):

(5) $$CRC=\frac{m_{c}-m_{0}}{m_{0}}$$

where CRC is the centrifuge retention capacity (g/g), $m_{c}$ is the mass of the gel after centrifugation (g), and $m_{0}$ is the dry weight of the sample (g).

#### 4.5.6. Absorption Under Load (AUL)

The AUL was measured using a customized setup based on Archimedes’ buoyancy principle, following the modified method of Islam et al. [57], with the test conditions detailed in Supplementary Information S1.3. In brief, 0.2000 g of dried hydrogel sample was sealed in a dialysis bag (MWCO 47 kDa, 30 mm width, 5 cm length) and subjected to a 320 g load in 50 mL of pre-heated (38 °C) swelling medium (0.9 wt% NaCl or deionized water) for 24 h. The swollen gel was drained on gauze without squeezing and weighed. The effective pressure was corrected for buoyancy, with the dialysis bag area being $1.5\times 10^{-3}\;m^{2}$. Based on Archimedes’ buoyancy principle, after deducting the buoyancy of the liquid displaced by the dialysis bag, the actual net force on the sample is calculated as $F_{net}=m\cdot g-\rho_{medium}\cdot g\cdot V_{displaced}$. Over an effective contact area of $1.5\times 10^{-3}{\;m}^{2}$, the actual effective pressure generated is approximately 1.11 kPa (see Table S2 for details). For the commercial SAPs, the reported AUL values for the representative products (SAP1, SAP4, and SAP6) were individually obtained from three independent measurements (*n* = 3) (see Table 2). A detailed description of the setup, the buoyancy correction derivation, and the force/pressure data are provided in the Supplementary Information (Figure S1 and Table S2). It should be noted that the customized dialysis bag method used in this study differs structurally from traditional AUL industry standards. The sealed dialysis membrane introduces inevitable mass-transfer resistance and imposes additional spatial confinement on the three-dimensional swelling of the gel. Therefore, this method is primarily intended to evaluate the swelling potential of materials within a highly confined microenvironment.

#### 4.5.7. Rheological Properties

Rheological measurements were performed on an Anton Paar MCR-302 rheometer (Anton Paar GmbH, Graz, Austria) at 25 °C using a parallel plate geometry (plate diameter: 25 mm; gap: 0.5 mm). The rheological tests used as-synthesized gels with a polymer content of approximately 5 wt% (determined by the drying method). Samples were equilibrated for 10 min before measurement under a constant normal force of 0.5 N using smooth parallel plates. During the test, a thin layer of silicone oil was coated on the sample surface to minimize water evaporation. Strain sweep measurements were conducted from 0.01% to 100% at 1 Hz to characterize the strain-dependent viscoelastic behavior. Based on the strain sweep results, a constant strain of 0.05% was selected for subsequent frequency sweep measurements over an angular frequency range of 0.1–100 rad s^−1^ to obtain the storage modulus (G′) and loss modulus (G″). The complex viscosity |η*| was calculated as $|\eta^{*}|=\frac{\sqrt{{\left(G^{\prime }\right)}^{2}+{\left(G^{\prime \prime }\right)}^{2}}}{\omega }$. The justification for the 0.05% strain selection is provided in the Supplementary Information (S1.4).

### 4.6. Statistical Analysis and Data Reproducibility Statement

To ensure the reliability of the core performance indicators during the formulation optimization process, quantitative data for absorption capacity, centrifuge retention capacity (CRC), and absorption under load (AUL) were obtained based on three replicate measurements from the same synthesized batch (*n* = 3) and are expressed as the mean ± standard deviation (SD). Structural and physical state characterizations (including rheology, thermogravimetric analysis, water retention testing, and cyclic swelling behavior) were performed on representative samples using single measurements. Commercially available SAP products, manufactured to industrial standards, possess extremely high batch stability and uniformity; thus, they served solely as single baseline references for evaluating the testing system in this study. Similarly, given the massive performance span observed between the composite and the pure polymer control group, the absolute performance differences between the groups far exceed the measured sample standard deviations. This intuitive macroscopic difference sufficiently demonstrates the statistical significance of the biomass scaffold’s reinforcing effect. Therefore, this study considers descriptive statistics sufficient to support the core conclusions, without introducing additional inferential statistical hypothesis testing. All data visualization and non-linear curve fitting were performed using OriginPro 2026 software (OriginLab Corporation, Northampton, MA, USA).

## Figures and Tables

**Table 1:** Standardized thermal degradation parameters obtained from the TGA and DTG curves for CBP, P(AA-co-AM) SAP, and P(AA-co-AM)/CBP SAP.

Parameter	CBP	P(AA-co-AM) SAP	P(AA-co-AM)/CBP SAP
$T_{5\%}$ (°C)	129.0	87.9	100.8
$T_{10\%}$ (°C)	157.1	165.4	169.7
$T_{onset}$ (°C)	156.4	336.4	380.5
$T_{max}$ (°C)	187.8	339.8	438.2

Note: $T_{5\%}$ and $T_{10\%}$ represent the temperatures corresponding to 5% and 10% mass loss, respectively. To ensure a scientifically rigorous comparison, the extrapolated onset decomposition temperature ($T_{onset}$, determined via the baseline-tangent intersection method) and the maximum degradation rate temperature ($T_{max}$) were extracted exclusively from the primary decomposition stage of the main network framework for all samples. The low-temperature events (e.g., dehydration, initial decomposition of labile components) and the high-temperature events (>700 °C, deep carbonization of residual char) were excluded from this evaluation, as they do not reflect the intrinsic thermal stability of the crosslinked network. This selection criterion is consistently applied across all samples to enable a valid comparative assessment.

**Table 2:** Comparison of free-swelling absorption performance in deionized water, 0.9 wt% NaCl solution, and synthetic urine, together with centrifuge retention capacity (CRC, measured in 0.9 wt% NaCl solution), for P(AA-co-AM)/CBP SAP, the control sample, and eleven commercial SAPs. For the synthesized SAPs, data are derived from three replicate measurements obtained from the same synthesized batch (*n* = 3). All commercial SAP samples were tested as received without secondary grinding or physical sieving. Prior to testing, all commercial SAP samples were strictly stored in sealed containers at room temperature.

Sample	Free-Swelling Absorption in Deionized Water (g/g)	Free-Swelling Absorption in 0.9 wt% NaCl (g/g)	Free-Swelling Absorption in Synthetic Urine (g/g)	CRC (g/g)
P(AA-co-AM)/CBP SAP	2801.00 ± 124.52	142.72 ± 8.22	65.04 ± 2.57	73.17 ± 3.11
P(AA-co-AM) SAP	721.14 ± 2.55	74.98 ± 0.30	44.81 ± 0.34	46.85 ± 3.68
SAP1	438.20	95.91	36.90	33.96
SAP2	575.39	90.87	37.23	35.78
SAP3	541.69	95.67	39.02	33.57
SAP4	618.98	91.13	35.67	37.87
SAP5	572.00	80.96	34.93	36.39
SAP6	538.64	89.36	51.87	33.53
SAP7	628.54	76.24	42.24	33.41
SAP8	463.34	79.41	37.79	30.07
SAP9	517.64	83.64	37.92	35.46
SAP10	396.82	76.52	37.27	36.58
SAP11	426.36	77.69	34.41	31.71

## Data Availability

The data presented in this study are available on request from the corresponding author due to a pending patent application.

## Supplementary Materials

The following supporting information can be downloaded at https://www.mdpi.com/article/10.3390/gels12090768/s1. Table S1: Detailed synthesis formulation parameters for P(AA-co-AM) SAP and P(AA-co-AM)/CBP SAP; Figure S1: Schematic diagram of the measurement system; Table S2: Applied force and pressure on the swelling SAP enclosed inside a dialysis tube segment at 38 °C in swelling media (deionized water and saline) at their default pH for 24 h; Table S3: Free-swelling absorption capacity of P(AA-co-AM)/CBP SAP prepared from replicate measurements; Table S4: Commercial SAPs used for comparison; Table S5: Detailed technical specifications and background information of the selected commercial SAP products; Figure S2: FTIR spectra of CBP, P(AA-co-AM) SAP, their physical mixture, and P(AA-co-AM)/CBP SAP. Marked peaks indicate the characteristic shifts (3435→3442 cm^−1^, 1558→1567 cm^−1^); Table S6: Non-linear pseudo-second-order kinetic parameters, statistical evaluation, and 95% confidence intervals for P(AA-co-AM)/CBP SAP and P(AA-co-AM) SAP; Figure S3: Residual analysis plots for the non-linear pseudo-second-order kinetic fitting of (a) P(AA-co-AM) SAP and (b) P(AA-co-AM)/CBP SAP. Each panel displays the regular residuals versus time (min) (top left), the histogram of regular residuals (top right), the regular residuals versus fitted values (predicted $Q_{t}$) (bottom left), and the normal probability plot of the residuals (bottom right); Table S7: Fickian diffusion parameters (initial stage, F < 0.6); Table S8: Contents of this Supporting Information.References [57, 65] are cited in the supplementary materials.
