# Supplementary material for: Cucumber Biomass Powder as Reinforcing Scaffold for Poly(acrylic acid-co-acrylamide) Superabsorbent Hydrogels: Structure and Properties
Source: Gels. 2026 Aug 27;12(9):768. doi: 10.3390/gels12090768 (PMC13606901; doi:10.3390/gels12090768)
Supplement: Supplementary file 1 [file gels-12-00768-s001.zip › gels-4494084-supplementary.pdf]

Supporting Information

Cucumber Biomass Powder as Reinforcing Scaffold for Poly(acrylic acid-co-acrylamide) Superabsorbent Hydrogels: Structure and Properties

S1. Detailed Experimental Methods

S1.1 Synthesis formulation and detailed procedures

The detailed formulation parameters for the optimized P(AA-co-AM)/CBP SAP and the CBP-free control sample are summarized in Table S1.

Table S1. Detailed synthesis formulation parameters for P(AA-co-AM) SAP and P(AA-co-AM)/CBP SAP.

Parameter / Reagent	P(AA-co-AM) SAP	P(AA-co-AM)/CBP SAP
Acrylic Acid (AA)	1.4400 g (20.0 mmol)	1.4400 g (20.0 mmol)
Acrylamide (AM)	0.7200 g (10.13 mmol)	0.7200 g (10.13 mmol)
Mass Ratio (AA:AM)	2 : 1	2 : 1
NaOH	0.5995 g (15.0 mmol)	0.5995 g (15.0 mmol)
CBP	0 g	0.1440 g (10 wt%)
MBA	0.576 mg (0.04 wt%)	0.576 mg (0.04 wt%)
APS	7.92 mg (0.55 wt%)	7.92 mg (0.55 wt%)
$NaHSO_3$	0.0036 g (0.25 wt%)	0.0036 g (0.25 wt%)
Total Water Volume	5.24 mL	5.24 mL
Reaction Temperature	70 °C	70 °C

Notes on experimental procedures: In Table S1, the mass fractions (wt%) of CBP, the crosslinker (MBA), and the initiators are all strictly calculated based on the mass of

acrylic acid (AA). The mass ratio of APS to $NaHSO_3$ was fixed at 2.2 : 1. Since the core focus of this study is the macroscopic water absorption and rheological properties of the purified gel networks in the swollen state, the initial/final pH of the system, absolute yield of the dry product, and gel fraction were not quantitatively measured.

During the pretreatment and polymerization stages, continuous magnetic stirring was applied. Although the rotational speed was not quantitatively recorded, a constant stirring rate was consistently maintained during the pretreatment and polymerization stages to ensure the homogeneous dispersion of the CBP scaffold and monomers in the aqueous phase. To ensure precise dosing of trace-level reagents and minimize weighing errors, the crosslinker (MBA) and redox initiators (APS and $NaHSO_3$) were added as 100 μL aliquots with a micropipette from pre-calibrated bulk aqueous stock solutions. The exact concentrations of these standardized stock solutions were formulated as 5.76 mg/mL for MBA, 79.2 mg/mL for APS, and 36.0 mg/mL for $NaHSO_3$. The redox initiator solutions (APS and $NaHSO_3$) were added dropwise sequentially over a span of approximately 1 minute. Following polymerization, the bulk hydrogel was sectioned into small fragments and subjected to an exhaustive washing protocol to extract unreacted monomers and soluble oligomers. Specifically, the gel pieces were immersed in approximately 30 mL of anhydrous ethanol and washed for 3 consecutive cycles (30 min per cycle) under gentle shaking, followed by drying in an oven at 75 °C.

S1.2 Proximate composition analysis of CBP

The proximate composition of the unrefined cucumber biomass powder (CBP) was determined by a certified third-party testing agency (Anhui Jingwei Testing Technology Co., Ltd.) strictly following the National Standards of the People's Republic of China (GB/T). The specific standard methods used are as follows:

Crude protein (17.70%): Determined according to GB/T 6432-2018.

Crude fat (3.5%): Determined according to GB/T 6433-2025.

Crude fiber (6.2%): Determined according to GB/T 6434-2022 (filter crucible method).

Moisture (11.0%): Determined according to GB/T 6435-2014.

Crude ash (8.9%): Determined according to GB/T 6438-2007.

S1.3 Absorption under load (AUL) using a customized setup

A self-designed Absorption Under Load (AUL) testing apparatus was fabricated according to Archimedes' buoyancy principle, following the modified experimental protocol reported by Islam et al. [S1].

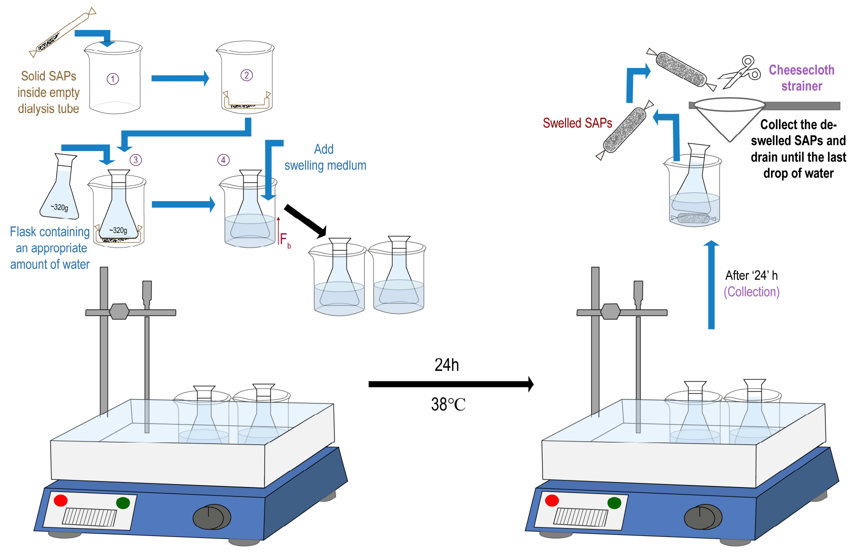

Figure S1. Schematic diagram of the measurement system.

Principle and buoyancy correction. The net force on the hydrogel is:

$$F_{net} = F_g - F_b = m \cdot g - \rho_{medium} \cdot g \cdot V_{displaced}$$

The effective pressure is $P = F_{net}/A$, where $A = 1.5\times10^{-3}$ m^2 (dialysis bag: 5 cm × 3 cm). A 320 g weight was used based on preliminary experiments confirming a measurable but not collapse-inducing pressure; 24 h was confirmed as sufficient to reach equilibrium under load.

Detailed procedure. A 0.2 g dried sample was sealed in a dialysis bag (MWCO 47 kDa, 30 mm width, 5 cm length) and placed at the bottom of a beaker. A 320 g weight was

placed on top, and 50 mL of pre-heated (38 °C) swelling medium (0.9 wt% NaCl or deionized water) was added. The beaker was kept in a 38 °C water bath for 24 h. After swelling, the gel was removed, drained on gauze without squeezing, and weighed.

Table S2. Applied force and pressure on the swelling SAP enclosed inside a dialysis tube segment at 38 °C in swelling media (deionized water and saline) at their default pH for 24 h.

Sample Name	Total weight (g)	Corrected force (N)	Corrected pressure (Pa)
P(AA-co-AM)/CBP SAP	320.7	1.671	1114.0
P(AA-co-AM) SAP	320.1	1.668	1112.4
Commercial SAP1	319.6	1.674	1116.1
Commercial SAP4	321.3	1.677	1118.1
Commercial SAP6	319.9	1.670	1113.3

S1.4 Rheological measurements: selection of test conditions

Strain sweep measurements were conducted from 0.01% to 100% at a constant frequency of 1 Hz to characterize the strain-dependent viscoelastic behavior, a common approach for probing nonlinear responses in complex materials [S2]. The G′ of both samples initially increased with strain before decreasing at higher strains (Figure 8a in the main text). Such strain-induced stiffening (i.e., absence of a classical linear viscoelastic region with constant G′) is commonly observed in physically crosslinked

or hydrogen-bonded hydrogels, where reversible non-covalent interactions provide structural adaptability under small deformations.

Based on the strain sweep results (Figure 8a in the main text), a constant strain of **0.05%** was selected for subsequent frequency sweep measurements for the following reasons: (i) 0.05% is well below the strain at which G′ reached its maximum value (2.43% for pure SAP and 3.64% for the composite), ensuring that the measurements were performed in a low-strain regime where the network structure remained largely intact; (ii) at 0.05% strain, the signal-to-noise ratio was sufficient for reliable data acquisition; and (iii) this strain is consistent with similar hydrogel rheology studies where the pre-yield region is used for frequency sweeps to avoid network disruption.

S1.5 Replicate measurements of CBP-based SAP

To assess the reproducibility of the measurements, three replicate samples were prepared from the same synthesis batch following the identical procedure. Each replicate was tested under the same optimized conditions (initiator 0.8 wt%, MBA 0.04 wt%, CBP 10 wt%, neutralization degree 75%, reaction temperature 70 °C) to measure the 30 min free-swelling absorption capacity in deionized water and 0.9 wt% NaCl solution.

Table S3. Free-swelling absorption capacity of P(AA-co-AM)/CBP SAP prepared from replicate measurements.

Replicate No.	Q_{30min} in DI water (g/g)	Q_{30min} in 0.9 wt% NaCl (g/g)
1	2928.96	142.22
2	2680.24	151.18

Replicate No.	Q_{30min} in DI water (g/g)	Q_{30min} in 0.9 wt% NaCl (g/g)
3	2793.81	134.76
**Mean ± SD**	2801.00±124.52	142.72±8.22

The small standard deviation values across the three replicate measurements indicate good reproducibility. The relative standard deviations (RSDs) of water absorption and saline absorption were calculated as 4.45% and 5.76%, respectively, confirming that the absorption measurements were reproducible.

S1.6 Commercial SAP details

Eleven commercial SAPs were tested. **Table S4** lists their identifiers and sources.

Table S4. Commercial SAPs used for comparison.

ID	Brand/type	Source
SAP1	WANICE® SAP 500	Wanhua Chemical Group Co., Ltd.
SAP2	WANICE® SAP 900	Wanhua Chemical Group Co., Ltd.
SAP3	Nuoer SAP NR-610S	Shandong Nuoer Biological Technology Co., Ltd.
SAP4	AQUA KEEP SA60N	Sumitomo Seika Chemicals Co., Ltd.
SAP5	TAISAP® GK402	Formosa Plastics Corporation
SAP6	Satellite SAP CR-710	Satellite Chemical Co., Ltd.
SAP7	SANWET® IM-33X	SDP Global Co., Ltd.
SAP8	LG SAP 810ND	LG Chem
SAP9	AQUALIC™ CA QX-W-1683	Nippon Shokubai Co., Ltd.
SAP10	HySorb® N 7059	BASF-YPC Company Limited
SAP11	HySorb® N 2330	BASF-YPC Company Limited

SAP1–SAP11 are leading imported brands (hygiene/diaper grade) obtained from local suppliers.

All samples were tested under identical conditions in our laboratory.

Table S5. Detailed technical specifications and background information of the selected commercial SAP products.

ID	Intended Application	Available Particle Size Distribution	Batch / Lot / Grade Info	Storage Conditions Prior to Testing
SAP1	Personal hygiene products (diapers, sanitary pads)	150–850 μm (Hygiene grade standard)	WANICE® SAP 500 / Commercial hygiene grade	As-received; sealed container at room temperature (25 °C, <60% RH)
SAP2	Personal hygiene products (high-retention core diapers)	150–850 μm (Hygiene grade standard)	WANICE® SAP 900 / Commercial hygiene grade	As-received; sealed container at room temperature (25 °C, <60% RH)
SAP3	Fast-speed SAP for composite core (top layer)	150–850 μm (Commercial standard)	Nuoer NR-610S / Commercial hygiene grade	As-received; sealed container at room temperature (25 °C, <60% RH)
SAP4	Feminine care and Ultra-thin diapers	500–850 μm (10.8%), 180–500 μm (86.8%), 106–180 μm (2.1%)	AQUA KEEP SA60N / Commercial grade	As-received; sealed container at room temperature (25 °C, <60% RH)
SAP5	Medical/Hygiene hydrogels (e.g., antipyretic paste) and specialty sheets	150–850 μm (Commercial standard)	TAISAP® GK402 / Commercial specialty grade	As-received; sealed container at room temperature (25 °C, <60% RH)
SAP6	Composite core (bottom layer) and	150–850 μm (Commercial standard)	Satellite CR-710 / Commercial	As-received; sealed container at room

	sanitary napkins		hygiene grade	temperature (25 °C, <60% RH)
SAP7	Personal hygiene and feminine care products	100–850 µm (Average: ~350–450 µm)	SANWET® IM-33X / Commercial hygiene grade	As-received; sealed container at room temperature (25 °C, <60% RH)
SAP8	High-performance absorbent cores (diapers)	150–850 µm (Commercial standard)	LG SAP 810ND / Commercial hygiene grade	As-received; sealed container at room temperature (25 °C, <60% RH)
SAP9	Daily hygiene products and diapers	Mean particle size: 300–450 µm	AQUALIC™ CA QX-W-1683 / Commercial grade	As-received; sealed container at room temperature (25 °C, <60% RH)
SAP10	Premium baby diapers and adult incontinence products	150–850 µm (≥93.5 wt%), >600µm (≤15.5 wt%)	HySorb® N 7059 / Commercial hygiene grade	As-received; sealed container at room temperature (25 °C, <60% RH)
SAP11	General hygiene products and absorbent cores	150–850 µm (Standard range)	HySorb® N 2330 / Commercial hygiene grade	As-received; sealed container at room temperature (25 °C, <60% RH)

Note: All commercial superabsorbent polymers (SAP1–SAP11) were purchased from regional suppliers as standard commercial-grade products.

1. Particle Size Distribution: Technical values presented in the table are derived from supplier Technical Data Sheets (TDS) or publicly available industrial specifications. All commercial samples were tested as-received without secondary grinding, sieving, or

fractionation to best reflect their performance in practical commercial absorbent cores.

2. Batch / Lot Information: Exact industrial lot numbers, internal crosslinking densities, and proprietary synthesis formulations are protected as trade secrets by the respective manufacturers and were not disclosed by the suppliers.

3. Storage Conditions: Upon receipt, all bulk samples were immediately stored in dry, airtight glass containers at ambient room temperature (25 °C) and $< 60\%$ RH to prevent moisture absorption prior to testing.

S2. Supplementary Characterization Data

S2.1 FTIR of physical mixture (key evidence for interfacial hydrogen bonding)

To confirm that the FTIR peak shifts in the composite arise from chemical interactions (hydrogen bonding) rather than simple physical mixing, a physical mixture of CBP and P(AA-co-AM) SAP (1:10 wt/wt) was prepared and analyzed.

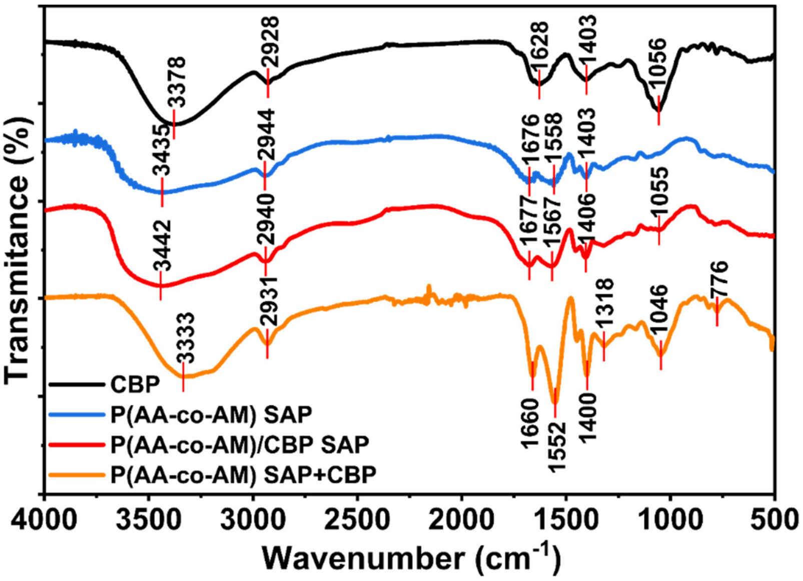

Figure S2. FTIR spectra of CBP, P(AA-co-AM) SAP, their physical mixture, and P(AA-co-AM)/CBP SAP. Marked peaks indicate the characteristic shifts (3435→3442 cm^{-1}, 1558→1567 cm^{-1}).

The FTIR spectrum of the physical mixture provides clear evidence for the absence of

chemical interactions between CBP and the P(AA-co-AM) network. Three characteristic peaks confirm this: the band at 1046 cm^{-1}, corresponding to the C–O/C–O–C stretching vibration of polysaccharides, originates from CBP (1056 cm^{-1}) and is retained in the physical mixture; the weak band at 776 cm^{-1}, which appears in pure P(AA-co-AM) SAP and the physical mixture, is not observed in CBP; and the small peak at 1318 cm^{-1}, a characteristic fingerprint of the P(AA-co-AM) network, is present in pure SAP and retained in the physical mixture but is absent in CBP. These features demonstrate that the physical mixture preserves the spectral signatures of both components without mutual influence.

The O–H/N–H stretching region provides additional insight. The physical mixture shows a broad envelope centered at approximately 3333 cm^{-1}, which is close to that of CBP (3378 cm^{-1}), indicating that the hydrogen-bonding environment in the physical mixture is dominated by the CBP component. In contrast, the chemically synthesized composite exhibits a distinct shift in this envelope to 3435 cm^{-1}, which is more comparable to pure P(AA-co-AM) SAP, suggesting that the CBP is integrated into the polymer network through interfacial interactions rather than simply coexisting as a separate phase.

In the fingerprint region, the physical mixture exhibits bands at 1660, 1552, and 1400 cm^{-1}, while the chemically synthesized P(AA-co-AM)/CBP SAP shows subtle but reproducible shifts to 1665, 1567, and 1406 cm^{-1}, respectively.

Since these shifts are absent in the physical mixture—despite the presence of both CBP-derived (1046 cm^{-1}) and SAP-derived (776 cm^{-1}) spectral markers—they cannot be attributed to simple spectral superposition. This provides spectroscopic evidence for both interfacial hydrogen bonding and potential partial chemical integration between CBP and the P(AA-co-AM) network. Notably, the shift in the polysaccharide C–O–C band from 1056 cm^{-1} (in pristine CBP) to approximately 1055 cm^{-1} (in the chemically synthesized composite), combined with the absence of any such shift in the physical mixture (where it remains at 1046 cm^{-1}), supports the occurrence of graft copolymerization. These findings are consistent with the proposed synthesis

mechanism (Section 2.1.1 of the main text), in which macro-oxygen radicals generated on the CBP surface initiate the graft polymerization of AA and AM monomers.

S3. Supplementary Performance Data

S3.1 Kinetic model fitting parameters

Table S6. Non-linear pseudo-second-order kinetic parameters, statistical evaluation, and 95% confidence intervals for P(AA-co-AM)/CBP SAP and P(AA-co-AM) SAP.

Sample	Parameter	Fitted Value	Standard Error	95% Confidence Interval
P(AA-co-AM)/CBP SAP	$Q_{e,cal}$ (g/g)	2930.34	49.93	[2822.47, 3038.20]
	$Q_{e,\text{exp}}$ (g/g)	2962.35		
	k ($g \cdot g^{-1} \cdot min^{-1}$)	4.21×10^{-4}	4.02×10^{-5}	[3.34×10^{-4}, 5.08×10^{-4}]
	R^2	0.9835		
P(AA-co-AM) SAP	$Q_{e,cal}$ (g/g)	736.35	9.12	[716.64, 756.05]
	$Q_{e,\text{exp}}$ (g/g)	739.96		
	k ($g \cdot g^{-1} \cdot min^{-1}$)	2.42×10^{-3}	1.71×10^{-4}	[2.05×10^{-3}, 2.79×10^{-3}]
	R^2	0.9891		

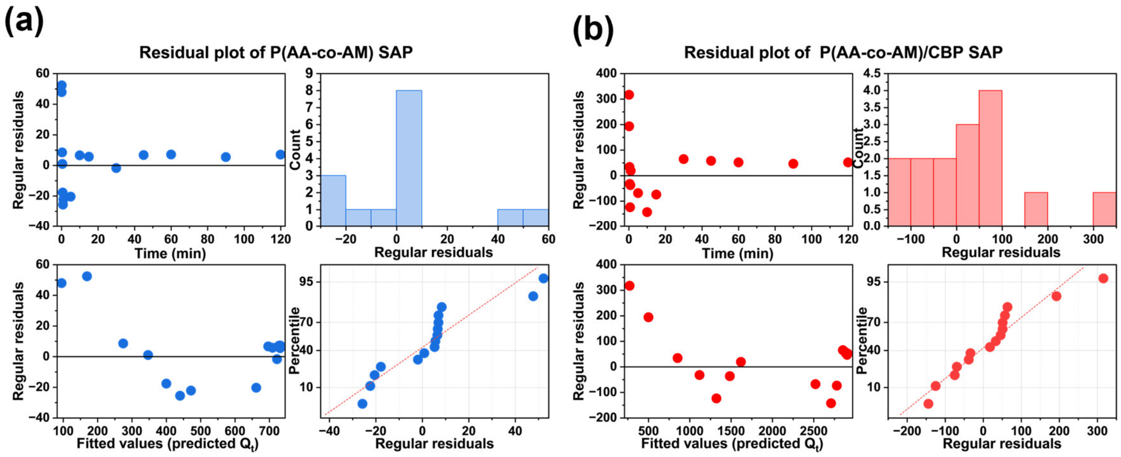

Figure S3. Residual analysis plots for the non-linear pseudo-second-order kinetic fitting of (a) P(AA-co-AM) SAP and (b) P(AA-co-AM)/CBP SAP. Each panel displays the regular residuals versus time (min) (top left), the histogram of regular residuals (top right), the regular residuals versus fitted values (predicted Q_t) (bottom left), and the normal probability plot of the residuals (bottom right).

Note: The random scatter of the residuals around the zero line and their approximate adherence to a normal distribution (as evidenced by the normal probability plots) confirm the validity and statistical reliability of the non-linear regression model without severe artificial variance weighting.

The Fickian diffusion model, $F = Q_t/Q_{eq} = k't^n$, was applied to the initial swelling stage ($F < 0.6$), where F is the fractional absorption at time t, k' is a structural parameter related to the polymer network, and n is the characteristic diffusion exponent. The exponent n indicates the transport mechanism: $n < 0.5$ corresponds to pseudo-Fickian (Case I) diffusion, $n = 0.5$ to ideal Fickian behavior, and $n > 0.5$ to non-Fickian transport. The fitting parameters are summarized in Table S7. The prime notation (k') is used to distinguish this structural parameter from the rate constant k in the pseudo-second-order kinetic model in the main text.

Table S7. Fickian diffusion parameters (initial stage, F < 0.6).

**Sample**	***n***	***k'* (min⁻ⁿ)**	***R²***	**Mechanism**
P(AA-co-AM) SAP	0.4524	0.627899	0.9885	Pseudo-Fickian ($n < 0.5$)
P(AA-co-AM)/CBP SAP	0.4063	0.504943	0.9664	Pseudo-Fickian ($n < 0.5$)

Both samples show $n < 0.5$, confirming pseudo-Fickian diffusion dominance in the early stage. The lower n value of the composite indicates a more pseudo-Fickian diffusion behavior, consistent with the perturbed chain mobility caused by the multicomponent biomass matrix and its open pore structure.

S4. Summary of Supporting Information

Table S8. Contents of this Supporting Information.

Section	Content
S1.1	Synthesis formulation and detailed procedures
S1.2	Proximate composition analysis of CBP
S1.3	AUL setup schematic, buoyancy correction, procedure, and pressure data
S1.4	Rheological measurements: selection of test conditions
S1.5	Replicate measurements of CBP-based SAP
S1.6	Commercial SAP details
S2.1	FTIR of physical mixture (evidence for interfacial hydrogen bonding)

Section	Content
S3.1	Kinetic model fitting parameters

References

[S1] M.S. Islam et al., Citric acid and cellulose derivatives-based superabsorbent hydrogels (SAH) as sustainable material alternatives for personal hygiene products, Chem. Eng. J. (2025) 170721. https://doi.org/10.1016/j.cej.2025.170721.

[S2] R.H. Ewoldt, A.E. Hosoi, G.H. McKinley, Nonlinear viscoelastic biomaterials: meaningful characterization and engineering inspiration, Integr. Comp. Biol. 49 (2009) 40–50. https://doi.org/10.1093/icb/icp009
